# DNA-based watermarks using the DNA-Crypt algorithm

**DOI:** 10.1186/1471-2105-8-176

**Published:** 2007-05-29

**Authors:** Dominik Heider, Angelika Barnekow

**Affiliations:** 1 Department of Experimental Tumorbiology, University of Muenster, Badestr. 9, D-48149 Muenster, Germany

## Abstract

**Background:**

The aim of this paper is to demonstrate the application of watermarks based on DNA sequences to identify the unauthorized use of genetically modified organisms (GMOs) protected by patents. Predicted mutations in the genome can be corrected by the DNA-Crypt program leaving the encrypted information intact. Existing DNA cryptographic and steganographic algorithms use synthetic DNA sequences to store binary information however, although these sequences can be used for authentication, they may change the target DNA sequence when introduced into living organisms.

**Results:**

The DNA-Crypt algorithm and image steganography are based on the same watermark-hiding principle, namely using the least significant base in case of DNA-Crypt and the least significant bit in case of the image steganography. It can be combined with binary encryption algorithms like AES, RSA or Blowfish. DNA-Crypt is able to correct mutations in the target DNA with several mutation correction codes such as the Hamming-code or the WDH-code. Mutations which can occur infrequently may destroy the encrypted information, however an integrated fuzzy controller decides on a set of heuristics based on three input dimensions, and recommends whether or not to use a correction code. These three input dimensions are the length of the sequence, the individual mutation rate and the stability over time, which is represented by the number of generations. *In silico *experiments using the Ypt7 in *Saccharomyces cerevisiae *shows that the DNA watermarks produced by DNA-Crypt do not alter the translation of mRNA into protein.

**Conclusion:**

The program is able to store watermarks in living organisms and can maintain the original information by correcting mutations itself. Pairwise or multiple sequence alignments show that DNA-Crypt produces few mismatches between the sequences similar to all steganographic algorithms.

## Background

Sensitive information, especially secret information must be protected against unauthorized access. To achieve this researchers have looked for new cryptographic or steganographic techniques. Existing algorithms encrypt or hide information in binary files, however there are other media, which can be used. There are several algorithms, which encode information into DNA sequences. Examples are the concepts of Clelland et al., Gehani et al., Leier et al, Wong et al. and Arita et al [1-5]. These techniques can be used for authentication or to store data for long time.

### Clelland et al

Inspired by the micro-dots used during the 2nd world war, Clelland et al. developed an extension of this principle [1]. The scientists produced artificial DNA strands, which contained secret messages. A triplet encodes one character or number (Table 1). The Clelland algorithm is a simple substitution cipher which encodes characters into DNA sequences using the following encoding function

**Table 1:** Clelland code table. Modified from Clelland et al. [1].

character = triplet	character = triplet
A = CGA	U = CTG
B = CCA	V = CCT
C = GTT	W = CCG
D = TTG	X = CTA
E = GGC	Y = AAA
F = GGT	Z = CTT
G = TTT	0 = ACT
H = CGC	1 = ACC
I = ATG	2 = TAG
J = AGT	3 = GCA
K = AAG	4 = GAG
L = TGC	5 = AGA
M = TCC	6 = TTA
N = TCT	7 = ACA
O = GGA	8 = AGG
P = GTG	9 = GCG
Q = AAC	⊔ = ATA
R = TCA	, = TCG
S = ACG	. = GAT
T = TTC	: = GCT

• *E *: *X *→ *Y*

• *X *∈ {*A*, *B*, *C*,..., *Z*, 0, 1,..., 9, ".",","," : ","⊔"}

• *Y *∈ {*xyz *: *x*, *y*, *z *∈ {*A*, *C*, *G*, *T*}}

The decoding function is corresponding *D *: *Y *→ *X*.

Now Clelland et al. ligated two primers with the synthesized DNA sequences, a forward and a reverse primer. These ligated sequences were mixed up with dummy strands. Important preconditions are:

• length of dummy strand = length of message DNA with primers

• #copies of each dummy = #copies of message DNA

The receiver must know the decoding function and the primer to decode the message. The primers are used for the polymerase chain reaction and in the last step the amplified DNA sequence has to be sequenced and decoded. To improve the security one can use dummy strands, which are not random but correspond to words out of a dictionary.

### Gehani et al

**Figure 1:**
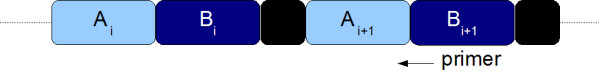
**DNA One-Time pad**. *A*_*i*_: plain text, *B*_*i*_: cipher text (and primer for the DNA polymerase), black box: stop Modified from Gehani et al. [2].

The original One-Time pad uses the XOR – exclusive **or **(⊕). In the case of DNA, the XOR is very impracticable and therefore it is better to use the properties of DNA. Gehani et al. established a DNA One-Time pad by creating word pairs [2]. The first word is the plain text and the second one is the cipher text. After such a block of plain and cipher text, there is a stop codon (Figure 1). The DNA polymerase completes the plain and cipher text.

To encode a message, the plain text is mixed with the DNA sequences. It binds directly to the corresponding complementary sequence. The DNA polymerase creates the cipher text accordingly and the decoding is functionally analogous. The cipher text binds to its complement and the DNA polymerase creates the plain text.

### Leier et al

**Figure 2:**
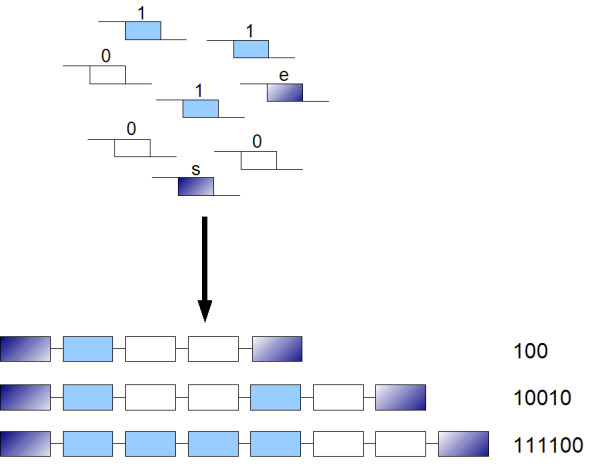
**DNA binary strands**. Short DNA strands represent the binary 1_2 _(light blue), 0_2 _(white), start and end marker (dark blue).These sequences can be ligated to long strands by using the sticky ends. Modified from Leier et al. [3].

Leier et al. encoded binary information into DNA sequences. A short DNA sequence represents the binary 1_2_, another one represents 0_2 _[3]. Further there are another two short DNA sequences, which represent start and end. The fragments have sticky ends and can be ligated (Figure 2). All resulting sequences are like this *s*{0_2_|1_2_}*e*. The start and end marker have primer sequences on one site for the polymerase chain reaction, which can not be ligated.

Although it seems to be more complicated, it is very similar to the algorithm of Clelland et al. The resulting DNA sequence is mixed with dummy strands and can only be detected and isolated knowing the primer sequences.

### Wong et al

Wong et al. developed a steganographic algorithm based on DNA, which is able to store data in living organisms [4]. The data are translated into a DNA sequence which is inserted into a vector. The insert sequence is flanked by two primer sequences which do not exist in the genome yet. This vector is introduced into a cell of a living organism where it coexists and is replicated with the genomic DNA. To extract the data they used a polymerase chain reaction.

Wong et al. used a substitution cipher similar to Clelland et al. to encode a song text into a DNA sequence and stored it in *Deinococcus radiodurans*. *Deinococcus radiodurans *survive extreme conditions, e.g. ionizing radiation, so the song text can be stored for hundreds of years.

### Arita et al

Arita et al. developed a steganographic algorithm based on the degenerative genetic code. Amino acid codes are redundant so that the translation of mRNA into proteins is a substitution cipher with the following characteristics

• *E *: *X *→ *Y*

• *X *∈ {*xyz *: *x*, *y*, *z *∈ {*A*, *C*, *G*, *U*}}

• *Y *∈ {*A*, *C*, *D*, *E*, *F*, *G*, *H*, *I*, *K*, *L*, *M*, *N*, *P*, *Q*, *R*, *S*, *T*, *V*, *W*, *Y*, *STOP*}

But the inverse function *D *: *Y *→ *X *is not injective.

An example:

threonine(T) = E(ACU) = E(ACC) = E(ACA) = E(ACG)

The triplet of threonine is redundant in the third base so mutations in the third base do not exert any influence on the translation of threonine and the translated protein. These mutations are called "synonymous substitutions", in contrast to the "non-synonymous substitutions".

Arita et al. translated each letter of the English alphabet into six codons (Table 2). A value of 0 means to keep the original base at the third position of a codon, while a value of 1 means to change the third base at that position. Arita et al. added a parity bit to each letter, to keep it odd for possible error detection [5]. They encoded 'KEIO' into the *ftsZ *gene of *Bacillus subtilis *which is essential for cell division and demonstrated as expected that the changed codon sequences did not affect the cell division, colony morphology, growth rate and sporulation frequency of these bacteria. To extract the encoded message one has to know the original sequence so that one can decide whether the codon is the original or the altered sequence.

**Table 2:** Arita et al. encryption. The translation table for the English alphabet. Modified from Arita et al. [5].

000001	...	010011	R
000010	E	100011	H
000100	T	001101	D
001000	A	010101	L
010000	O	100101	C
100000	S	011001	M
000111	N	101001	U
001110	F	110100	J
010110	G	111000	Q
011010	W	011111	Z
011100	Y	101111	'
100110	B	110111	.
101010	V	111011	&
101100	K	110001	P
001011	I	111101	
110010	X	111110	

### Comparison to DNA-Crypt

Clelland et al., Gehani et al. and Leier et al. produced synthesized DNA sequences which were mixed with dummy strands. These sequences contained a secret message. Knowing the unique primer sequence, the secret message can be read out.

Wong et al. and Arita et al. introduced DNA sequences containing a secret message into living organisms. Wong et al. used a vector which incorporated into the genome of *Deinococcus radiodurans *and Arita et al. used point mutations in redundant codons. Arita et al. used a parity bit for error detection. The disadvantage is that if mutations occur, the hidden information is lost.

**Figure 3:**
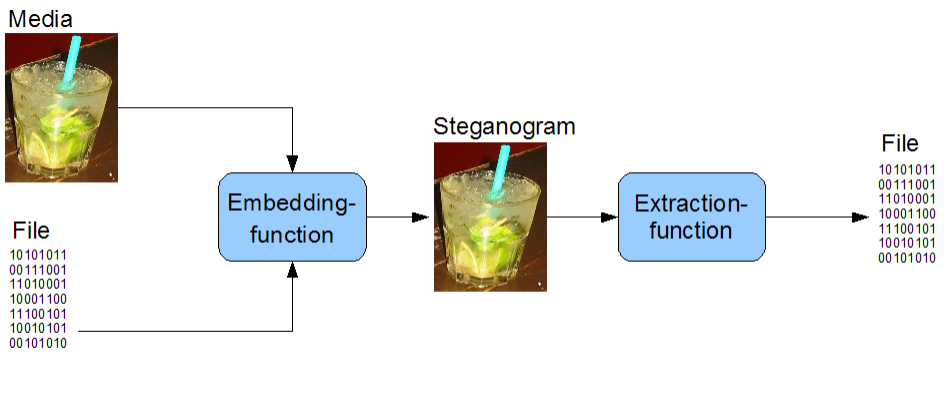
**steganographic algorithms**. The function of steganographic algorithms.

The DNA-Crypt algorithm is based on small redundant regions comparable to least significant bits in the case of image steganography (Figure 3). The least significant bits encode a difference in colour of just one on the colour scale, not visible to the human eye, and can be used to hide information in images.

Text or binary information can also be encoded using any DNA based encryption. However unlike image steganography, the DNA steganography does not lead to a loss of information if the focused range is a protein coding region. DNA-Crypt checks for "synonymous codons" in a genome and point mutations are produced by changing the bases [see Additional file 1].

**Figure 4:**
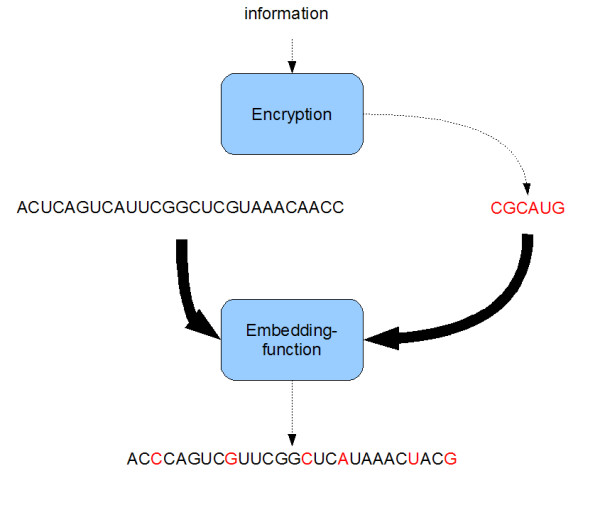
**The DNA-Crypt algorithm**. The function of the DNA-Crypt algorithm.

This algorithm offers the possibility to incorporate data into the genome of living organisms, using an alternative method to Wong et al. [4] (Figure 4). The algorithm is similar to the algorithm of Arita et al., but DNA-Crypt has some important extensions e.g. the use of several encryption and mutation correction codes, which allows encoding of binary information. These extensions are described in the next subsections [5]. A comparative overview of the algorithms and their features is shown in table 3.

**Table 3:** Comparison of the DNA encryption algorithms

algorithm	organism	affect	error detection	error correction	binary	encryption	utilization
Clelland et al.	-	-	-	-	-	-	20
Gehani et al.	-	-	-	-	-	-	-
Leier et al.	-	-	-	-	+	-	≤ 9
Wong et al.	+	+	+	+	-	-	20
Arita et al.	+	-	+	-	-	-	≤ 5
**DNA-Crypt**	+	-	+	+	+	+	≤ **8**

organism: the use of this algorithm in living organisms;

affect: observation that the algorithm exerts an effect on the organims;

error detection/correction: the algorithm shows an error detection/correction function;

binary: binary information can be encoded;

encryption: the use of binary encryption algorithm like AES or RSA;

utilization: storage utilization in a 100 bp DNA sequence;

- = negative;

+ = positive;

### Encoding binary information using DNA-Crypt

DNA-Crypt encodes binary information using the following substitution cipher:

• *E *: *X *→ *Y*

• *X *= {*xy *: *x, y *∈ {0_2_, 1_2_}}

• *Y *∈ {*A*, *C*, *G*, *T*}

A standard setting is given in table 4.

**Table 4:** DNA-Crypt binary encryption. The standard settings for encoding binary sequences in DNA-Crypt.

binary sequence	base
00_2_	T
01_2_	G
10_2_	C
11_2_	A

The binary sequence 0111001001001111_2 _would be encoded to *E*(0111001001001111_2_) = *GATCGTAA*.

Two bits could be encoded by one base, so one byte needs four bases for its encoding.

Based on this binary encryption, several private and public key cryptographic algorithms are integrated in DNA-Crypt:

• One-Time pad [6]

• AES [7]

• Blowfish [6]

• RSA [8,9]

To use DNA-Crypt one has to register so that DNA-Crypt can create AES, Blowfish and RSA keys for the user. These keys can be used to encrypt the binary information which then gets integrated into the genome. In addition it is possible to export and to import these keys and to exchange them with other users. Further the user can create new keys in DNA-Crypt or delete old ones. Another possibility is to use a One-Time pad instead of an encryption key. Compared to Arita et al. our substitution cipher allows to use several encryption algorithms as decribed above. In addition DNA-Crypt offers a better storage utilization compared to the algorithm of Arita et al. (four instead of six synonomous codons per character).

### Mutation correction

Mutations do not occur very often, approximately 10^-10 ^to 10^-15 ^per cell division, but they can destroy the encrypted information in DNA sequences. To correct these failures DNA-Crypt uses a correction code based on binary correction. One of them is the 8/4 Hamming-code and another one is the WDH-code [10]. The advantage of the WDH-code is that it can correct more mutations than the 8/4 Hamming-code. The n-times WDH-code repeats the enrypted DNA sequence n times. It can correct ⌊n−12⌋
 MathType@MTEF@5@5@+=feaafiart1ev1aaatCvAUfKttLearuWrP9MDH5MBPbIqV92AaeXatLxBI9gBaebbnrfifHhDYfgasaacH8akY=wiFfYdH8Gipec8Eeeu0xXdbba9frFj0=OqFfea0dXdd9vqai=hGuQ8kuc9pgc9s8qqaq=dirpe0xb9q8qiLsFr0=vr0=vr0dc8meaabaqaciaacaGaaeqabaqabeGadaaakeaadaGbdaqaamaalaaabaGaemOBa4MaeyOeI0IaeGymaedabaGaeGOmaidaaaGaayj84laawUp+aaaa@3608@ failures. All WDH-codes where n is an odd number are perfect.

The 8/4 Hamming-code can only correct ≤ 25% of the mutations. Four bits are used for information (b3,b2,b1,b0) and the other four bits as parity bits. A complete byte is represented by these eight bits *b*_3_,*b*_3 _⊕ *b*_2 _⊕ *b*_1_,*b*_2_, ¬*b*_2 _⊕ *b*_1 _⊕ *b*_0_,*b*_1_, ¬*b*_3 _⊕ *b*_1 _⊕ *b*_0_,*b*_0_, ¬*b*_3 _⊕ *b*_2 _⊕ *b*_0_

which are called h7, h6, h5, h4, h3, h2, h1, h0. To decode the byte, the following parity sums are build:

• *p *= *h*_7 _⊕ *h*_6 _⊕ *h*_5 _⊕ *h*_4 _⊕ *h*_3 _⊕ *h*_2 _⊕ *h*_1 _⊕ *h*_0_

*c*_0 _= *h*_7 _⊕ *h*_5 _⊕ *h*_1 _⊕ *h*_0_

*c*_1 _= *h*_7 _⊕ *h*_3 _⊕ *h*_2 _⊕ *h*_1_

*c*_2 _= *h*_5 _⊕ *h*_4 _⊕ *h*_3 _⊕ *h*_1_

If *p *= 1 there are 0 or 2 failures in the byte. The byte was transmitted correct, if the parity bits *c*_0_, *c*_1_, *c*_2 _are correct, which means equal to 1. If not, there happened 2 failures, which cannot be corrected.

If *p *= 0 there is 1 failure in the byte which can be corrected using table 5.

**Table 5:** The 8/4 Hamming-code correction table

*c*_0_	*c*_1_	*c*_2_	failure
1	1	1	*h*_6_
1	1	0	*h*_4_
1	0	1	*h*_2_
0	1	1	*h*_0_
0	0	1	*h*_7_
0	1	0	*h*_5_
1	0	0	*h*_3_
0	0	0	*h*_1_

Only one of four bits can be corrected. But not all mutations can be corrected by the 8/4 Hamming-code. Failures which only differ in one bit can be corrected, e.g. 00 ↔ 01 or 11 ↔ 10. Failures like 00 ↔ 11 or 10 ↔ 01 cannot be corrected.

The limiting resource for mutation correction is not the time, but the space. The advantage of the 8/4 Hamming-code is that it is very compact. The space requirements of the 8/4 Hamming-code is *f*(*n*) = 2*n *∈ Θ(*n*). In contrast to f(n)={n2, n is an odd numbern2+n, else}∈Θ(n2)
 MathType@MTEF@5@5@+=feaafiart1ev1aaatCvAUfKttLearuWrP9MDH5MBPbIqV92AaeXatLxBI9gBaebbnrfifHhDYfgasaacH8akY=wiFfYdH8Gipec8Eeeu0xXdbba9frFj0=OqFfea0dXdd9vqai=hGuQ8kuc9pgc9s8qqaq=dirpe0xb9q8qiLsFr0=vr0=vr0dc8meaabaqaciaacaGaaeqabaqabeGadaaakeaacqWGMbGzcqGGOaakcqWGUbGBcqGGPaqkcqGH9aqpdaGadeqaauaabeqaceaaaeaacqWGUbGBdaahaaWcbeqaaiabikdaYaaakiabcYcaSiabbccaGiabd6gaUjabbccaGiabdMgaPjabdohaZjabbccaGiabdggaHjabd6gaUjabbccaGiabd+gaVjabdsgaKjabdsgaKjabbccaGiabd6gaUjabdwha1jabd2gaTjabdkgaIjabdwgaLjabdkhaYbqaaiabd6gaUnaaCaaaleqabaGaeGOmaidaaOGaey4kaSIaemOBa4MaeiilaWIaeeiiaaIaemyzauMaemiBaWMaem4CamNaemyzaugaaaGaay5Eaiaaw2haaiabgIGiolabfI5arjabcIcaOiabd6gaUnaaCaaaleqabaGaeGOmaidaaOGaeiykaKcaaa@620B@ for the WDH-code.

For example to encode one byte, which means a DNA sequence of four bases, the 8/4 Hamming-code needs eight synonymous codons instead of twenty synonymous codons for the 5-times WDH-code. In contrast to the data published by Arita et al. we can not only exibit error detection but error corrections which enables us to maintain the data. This obviously represents an important advantage.

### fuzzy controller

**Figure 5:**
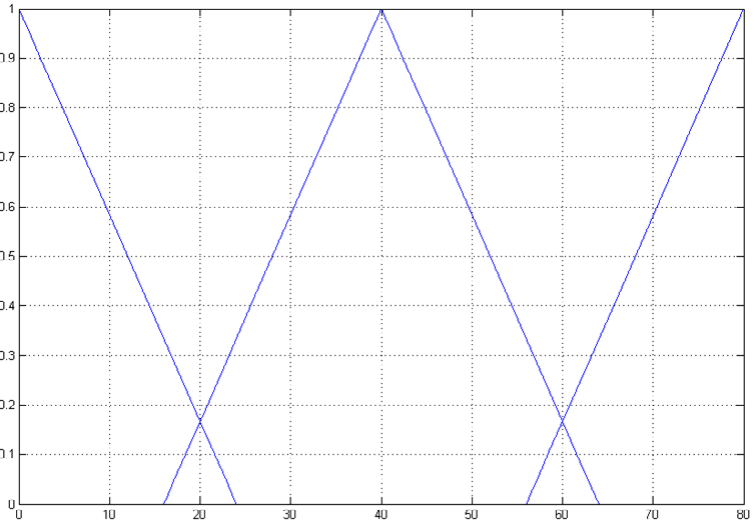
**The first input dimension**. The first input dimension of the fuzzy controller is the mutation rate. The first input dimension is separated intro three triangular sets *X*_*i *_= (*a*_*m*_, *a*_*λ*_, *a*_*ρ*_). The first called "*low*" = (0, 0, 6) describes a low mutation rate. The second "*middle*" = (10, 4, 16) and the third "*high*" = (20, 14, 20) describe a middle and a high mutation rate.

The integrated fuzzy controller decides and recommends whether to use the 8/4 Hamming-code, the WDH-code or no mutation correction for optimal performance [11-14] [see Additional file 2]. It uses the Singleton-fuzzyfication and has three input dimensions with each separated into three triangular sets. The first dimension is the individual mutation rate (*φ*) of the DNA sequence containing the secret message (Figure 5). This is based on a standard mutation rate, by default 1 * 10^-7 ^for prokaryotes and 1 * 10^-10 ^for eukaryotes, which is changed by specific mutation rates (*α*_*i*_) for each base pair. These changes are based on the transversion and transition rate and in addition on the stability (*δ*) of GC rich regions.

• φ=∑i=03αi/4
 MathType@MTEF@5@5@+=feaafiart1ev1aaatCvAUfKttLearuWrP9MDH5MBPbIqV92AaeXatLxBI9gBaebbnrfifHhDYfgasaacH8akY=wiFfYdH8Gipec8Eeeu0xXdbba9frFj0=OqFfea0dXdd9vqai=hGuQ8kuc9pgc9s8qqaq=dirpe0xb9q8qiLsFr0=vr0=vr0dc8meaabaqaciaacaGaaeqabaqabeGadaaakeaaiiGacqWFgpGzcqGH9aqpdaaeWaqaaiab=f7aHnaaBaaaleaacqWGPbqAaeqaaOGaei4la8IaeGinaqdaleaacqWGPbqAcqGH9aqpcqaIWaamaeaacqaIZaWma0GaeyyeIuoaaaa@3ABE@

• α0=#C∗(∑i=A,G,T3αCi/3)/δ
 MathType@MTEF@5@5@+=feaafiart1ev1aaatCvAUfKttLearuWrP9MDH5MBPbIqV92AaeXatLxBI9gBaebbnrfifHhDYfgasaacH8akY=wiFfYdH8Gipec8Eeeu0xXdbba9frFj0=OqFfea0dXdd9vqai=hGuQ8kuc9pgc9s8qqaq=dirpe0xb9q8qiLsFr0=vr0=vr0dc8meaabaqaciaacaGaaeqabaqabeGadaaakeaaiiGacqWFXoqydaWgaaWcbaGaeGimaadabeaakiabg2da9iabcocaJiabdoeadjabgEHiQiabcIcaOmaaqadabaGae8xSde2aaSbaaSqaaiabdoeadjabdMgaPbqabaGccqGGVaWlcqaIZaWmcqGGPaqkcqGGVaWlcqWF0oazaSqaaiabdMgaPjabg2da9iabdgeabjabcYcaSiabdEeahjabcYcaSiabdsfaubqaaiabiodaZaqdcqGHris5aaaa@47FE@

• α1=#G∗(∑i=A,C,T3αGi/3)/δ
 MathType@MTEF@5@5@+=feaafiart1ev1aaatCvAUfKttLearuWrP9MDH5MBPbIqV92AaeXatLxBI9gBaebbnrfifHhDYfgasaacH8akY=wiFfYdH8Gipec8Eeeu0xXdbba9frFj0=OqFfea0dXdd9vqai=hGuQ8kuc9pgc9s8qqaq=dirpe0xb9q8qiLsFr0=vr0=vr0dc8meaabaqaciaacaGaaeqabaqabeGadaaakeaaiiGacqWFXoqydaWgaaWcbaGaeGymaedabeaakiabg2da9iabcocaJiabdEeahjabgEHiQiabcIcaOmaaqadabaGae8xSde2aaSbaaSqaaiabdEeahjabdMgaPbqabaGccqGGVaWlcqaIZaWmcqGGPaqkcqGGVaWlcqWF0oazaSqaaiabdMgaPjabg2da9iabdgeabjabcYcaSiabdoeadjabcYcaSiabdsfaubqaaiabiodaZaqdcqGHris5aaaa@4808@

• α2=#A∗∑i=C,G,T3αAi/3
 MathType@MTEF@5@5@+=feaafiart1ev1aaatCvAUfKttLearuWrP9MDH5MBPbIqV92AaeXatLxBI9gBaebbnrfifHhDYfgasaacH8akY=wiFfYdH8Gipec8Eeeu0xXdbba9frFj0=OqFfea0dXdd9vqai=hGuQ8kuc9pgc9s8qqaq=dirpe0xb9q8qiLsFr0=vr0=vr0dc8meaabaqaciaacaGaaeqabaqabeGadaaakeaaiiGacqWFXoqydaWgaaWcbaGaeGOmaidabeaakiabg2da9iabcocaJiabdgeabjabgEHiQmaaqadabaGae8xSde2aaSbaaSqaaiabdgeabjabdMgaPbqabaGccqGGVaWlcqaIZaWmaSqaaiabdMgaPjabg2da9iabdoeadjabcYcaSiabdEeahjabcYcaSiabdsfaubqaaiabiodaZaqdcqGHris5aaaa@43C6@

• α3=#T∗∑i=A,C,G3αTi/3
 MathType@MTEF@5@5@+=feaafiart1ev1aaatCvAUfKttLearuWrP9MDH5MBPbIqV92AaeXatLxBI9gBaebbnrfifHhDYfgasaacH8akY=wiFfYdH8Gipec8Eeeu0xXdbba9frFj0=OqFfea0dXdd9vqai=hGuQ8kuc9pgc9s8qqaq=dirpe0xb9q8qiLsFr0=vr0=vr0dc8meaabaqaciaacaGaaeqabaqabeGadaaakeaaiiGacqWFXoqydaWgaaWcbaGaeG4mamdabeaakiabg2da9iabcocaJiabdsfaujabgEHiQmaaqadabaGae8xSde2aaSbaaSqaaiabdsfaujabdMgaPbqabaGccqGGVaWlcqaIZaWmaSqaaiabdMgaPjabg2da9iabdgeabjabcYcaSiabdoeadjabcYcaSiabdEeahbqaaiabiodaZaqdcqGHris5aaaa@43EE@

The first input dimension is separated into three triangular sets *X*_*i *_= (*a*_*m*_, *a*_*λ*_, *a*_*ρ*_) [15-20]. The first called "*low*" = (0, 0, 6) describes a low mutation rate. The second "*middle*" = (10, 4, 16) and the third "*high*" = (20, 14, 20) describes a middle and a high mutation rate.

**Figure 6:**
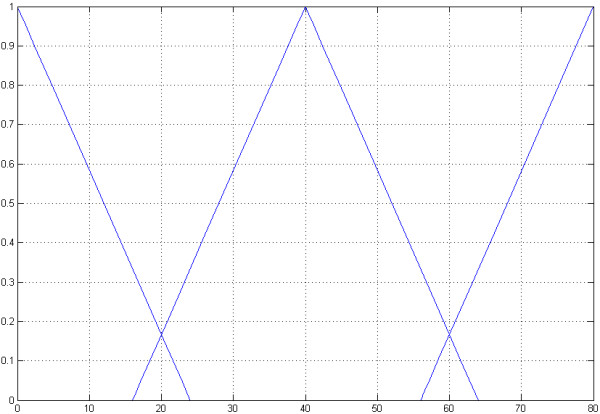
**The second input dimension**. The second input dimension of the fuzzy controller is the length of the sequence containing the encrypted message. The triangular sets are "*short*" = (0, 0, 24), "*middle*" = (40, 16, 64) and "*long*" = (80, 56, 80).

The second input dimension is the length of the DNA sequence containing the secret message (Figure 6).

The triangular sets are "*short*" = (0, 0, 24), "*middle*" = (40, 16, 64) and "*long*" = (80, 56, 80).

**Figure 7:**
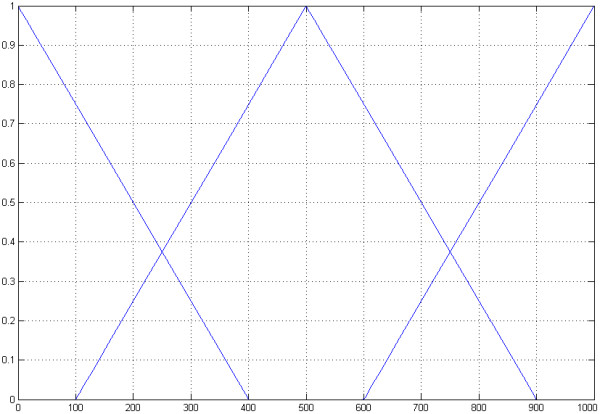
**The third input dimension**. The third input dimension is the stability over time, which is represented by the number of generations. It is separated into "*low*" = (0, 0, 400), "*middle*" = (500, 100, 900) and "*high*" = (1000, 600, 1000).

The third input dimension is the stability over time, which is represented by the number of generations (Figure 7). It is separated into "*low*" = (0, 0, 400), "*middle*" = (500, 100, 900) and "*high*" = (1000, 600, 1000).

**Figure 8:**
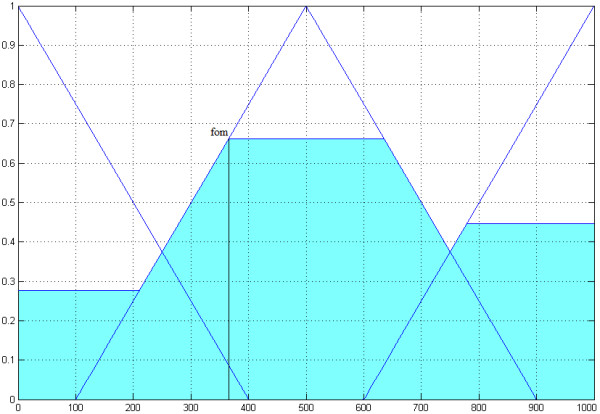
**The output dimension of the fuzzy controller**. The triangular sets are "*none*" = (0, 0, 400), "*Hamming – code*" = (500, 100, 900) and "*WDH – code*" = (1000, 600, 1000). The maximum of a triangular set, calculated by the set of heuristics of the fuzzy controller, means a cut on the y axis. A cut at 0.28 for none correction code, at 0.67 for Hamming-code and at 0.45 for the WDH-code is shown. The first-of-maximum (fom) represents the recommended correction code, in this case the fuzzy controller recommends the Hamming-code.

The three input dimensions are linked through a set of rules based on heuristics to one output dimension [see Additional file 3]. The maximum of each correction code means a cut on the y axis (Figure 8). In the next step the fuzzy controller decides, whether to use an 8/4 Hamming-code, a WDH-code or no mutation correction by using the first-maximum method and recommends it to the user.

## Results

The program described above was tested by *in silico *experiments using the DNA sequence encoding the Ypt7 in *Saccharomyces cerevisiae*.

### Ypt7

The small GTPases termed Ypt in yeast and Rab in higher eukaryotes are molecular switches in cellular transport processes [21]. Each Ypt protein is localized to the membrane of specific intracellular compartments and highly specific for a particular transport step [22].

The Ypt7 GTPase from *S. cerevisiae *is involved in late endosome-to-vacuole transport and vacuole fusion events [23,24]. Ypt7 is one of the 11 members of the *S. cerevisiae *Ypt family and is homologous to mammalian Rab7.

Analysis of the Ypt7 DNA sequence showed that 32% of the codons allow synonymous substitutions, resulting in 16 bytes, which could be encrypted (Table 6). The first steganogram contains the message "this is a test" and the second one "yet another test" [see Additional file 4].

**Table 6:** DNA-Crypt analysis of the Ypt7 DNA sequence. It is possible to encrypt 16.5 bytes in the Ypt7 DNA sequence.

name	amino acids	syn. codons	bytes
Ypt7	206	32.039%	16.5

The results of the analyses of these steganograms with the fuzzy controller are shown in table 7. Translation with DNA-Crypt and the *Expasy Translate Tool *shows that the translated amino acid sequences are identical [25].

**Table 7:** Ypt7 steganogram fuzzy controller results

name	*φ*	correction code
steganogram 1	9.48	8/4 Hamming-code
steganogram 2	10.5	8/4 Hamming-code

**Figure 9:**
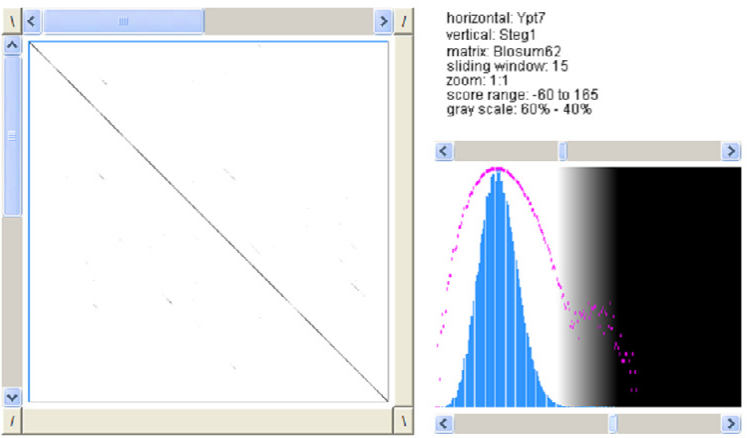
**Dotplot of Ypt7 and steganogram 1**. Pairwise sequence alignment with *Dotlet *between the original sequence and the steganogram containing "this is a test" [26].

**Figure 10:**
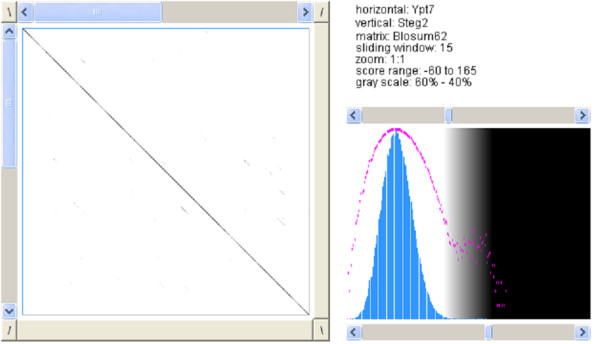
**Dotplot of Ypt7 and steganogram 2**. Pairwise sequence alignment with *Dotlet *between the original sequence and the steganogram containing "yet another test" [26].

**Figure 11:**
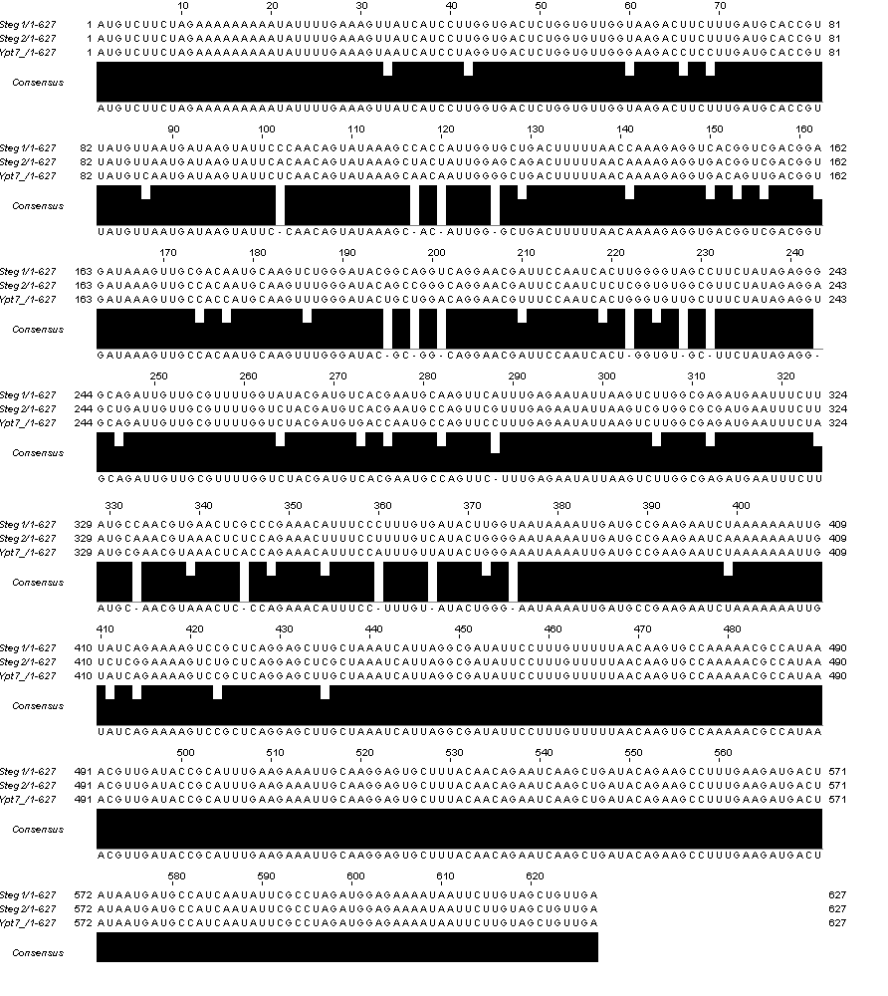
**Multiple sequence alignment**. Multiple sequence alignment of the original sequence and two steganograms [27].

The pairwise and the multiple sequence alignments show a few mismatches between the three sequences (Figures 9, 10, 11).

The pairwise sequence alignment was performed with *Dotlet *and the multiple sequence alignment was performed using *ClustalW *of the *European Bioinformatics Institute *with standard settings [26,27].

## Discussion

DNA-Crypt produces few sequence mismatches similar to the low noise in image steganography. In case of image steganography one can look at the least significant bits to attack the steganographic algorithms. To attack DNA steganography one can perform pairwise or multiple sequence alignments with the original sequences.

## Conclusion

The DNA-Crypt algorithm can encode cryptic messages into DNA sequences, which can be used as watermarks for authentication. DNA-Crypt is a substantial extension to other steganographic algorithms based on DNA, which can be used in combination with a binary encryption algorithm such as AES, RSA or Blowfish and a mutation correction code such as the Hamming-code or the WDH-code. The most appropriate code of these correction codes can be selected by a fuzzy controller, which uses three input dimensions.

Mutations, which cause changes in the reading frame, are problematic and are not appropriate for DNA steganography. Mutations, which change a non-synonymous codon to a synonymous codon or vice versa are more important as these mutations cause errors in the encrypted information. The relevance of these errors depends on the encrypted information. If the encrypted information is an image, e.g. a logo, there would be only a linear colour shift in the image, which is not very relevant and can be corrected very easily. However if the encrypted information must remain correct, e.g. a password, the WDH-code must be used to detect these mutations.

We have not encoutered any problems so far performing our *in silico *analyses using DNA-Crypt watermarks in DNA coding regions. The use of DNA-Crypt in non-coding sequences like a regulatory RNA sequence or promoter, and enhancer sequences has to be tested *in silico *and *in vivo*. Further analyses to clarify, whether alternative splicing events pose a problem for watermarks still have to be carried out. In conclusion DNA-Crypt algorithm represents an interesting tool for hiding authenticating watermarks within coding DNA sequences *in silico *and most probably in living organisms without affecting the process of protein translation and protein function.

## Availability and requirements

**Project Name: **DNA-Crypt

**Project Homepage: **

**Operating Systems: **Cross-platform

**Programming Language: **Java 5.0 or higher

## Authors' contributions

DH: conception, software development, sequence alignments, figure preparation, manuscript preparation

AB: conception, design, manuscript preparation, coordination, research funds collection. The authors read and approved the final manuscript.

## Supplementary Material

Additional file 1 The DNA-Crypt v.2. Click here for file

Additional file 2 The fuzzy controller. Click here for file

Additional file 3 The set of rules for the fuzzy-controller. Click here for file

Additional file 4 The DNA sequences of the Ypt7 and the two steganograms. Click here for file
