# Supplementary material for: DNA-based watermarks using the DNA-Crypt algorithm
Source: BMC Bioinformatics. 2007 May 29;8:176. doi: 10.1186/1471-2105-8-176 (PMC1904243; doi:10.1186/1471-2105-8-176)
Supplement: Additional file 1 — The DNA-Crypt v.2. [file 1471-2105-8-176-S1.zip › help/doc/allclasses-frame.html]

All Classes


**All Classes**
  

|  |
| --- |
| AES   AminoSteg   Analyser   BitCoding   Blowfish   BrowserControl   Clelland   *CorrectionCode*   DNACrypt   ForeignAESBlowfishKey   *ForeignKey*   ForeignRSAKey   GenomeOperator   HammingCode   KeyManager   NonCorrection   OneTimePad   RSA   User   UserManager   WDHC |
