# Supplementary material for: DNA-based watermarks using the DNA-Crypt algorithm
Source: BMC Bioinformatics. 2007 May 29;8:176. doi: 10.1186/1471-2105-8-176 (PMC1904243; doi:10.1186/1471-2105-8-176)
Supplement: Additional file 1 — The DNA-Crypt v.2. [file 1471-2105-8-176-S1.zip › help/doc/asymmetric/RSA.html]

RSA


|  |  |  |  |  |  |  |  |  |  |  |
| --- | --- | --- | --- | --- | --- | --- | --- | --- | --- | --- |
| |  |  |  |  |  |  |  |  | | --- | --- | --- | --- | --- | --- | --- | --- | | **Overview** | **Package** | **Class** | **Use** | **Tree** | **Deprecated** | **Index** | **Help** | | |  |
| PREV CLASS   NEXT CLASS | **FRAMES**    **NO FRAMES**     **All Classes** |
| SUMMARY: NESTED | FIELD | CONSTR | METHOD | DETAIL: FIELD | CONSTR | METHOD |


---


## asymmetric Class RSA

```
java.lang.Object
  asymmetric.RSA
```

---

``` public class RSA extends java.lang.Object ```

The Class represents a RSA keygenerator.

**Author:**
:   Dominik Heider

---

| **Constructor Summary** | |
| --- | --- |
| `RSA()`             creates a secret key pair for the RSA algorithm. |


| **Method Summary** | |
| --- | --- |
| `java.security.PrivateKey` | `getPrivate()` |
| `java.security.PublicKey` | `getPublic()` |

| **Methods inherited from class java.lang.Object** |
| --- |
| `equals, getClass, hashCode, notify, notifyAll, toString, wait, wait, wait` |

| **Constructor Detail** |
| --- |

### RSA

```
public RSA()
```

:   creates a secret key pair for the RSA algorithm.


| **Method Detail** |
| --- |

### getPrivate

```
public java.security.PrivateKey getPrivate()
```

:   **Returns:**: the Private Key

---


### getPublic

```
public java.security.PublicKey getPublic()
```

:   **Returns:**: the Public Key


---


|  |  |  |  |  |  |  |  |  |  |  |
| --- | --- | --- | --- | --- | --- | --- | --- | --- | --- | --- |
| |  |  |  |  |  |  |  |  | | --- | --- | --- | --- | --- | --- | --- | --- | | **Overview** | **Package** | **Class** | **Use** | **Tree** | **Deprecated** | **Index** | **Help** | | |  |
| PREV CLASS   NEXT CLASS | **FRAMES**    **NO FRAMES**     **All Classes** |
| SUMMARY: NESTED | FIELD | CONSTR | METHOD | DETAIL: FIELD | CONSTR | METHOD |


---
