# Supplementary material for: DNA-based watermarks using the DNA-Crypt algorithm
Source: BMC Bioinformatics. 2007 May 29;8:176. doi: 10.1186/1471-2105-8-176 (PMC1904243; doi:10.1186/1471-2105-8-176)
Supplement: Additional file 1 — The DNA-Crypt v.2. [file 1471-2105-8-176-S1.zip › help/doc/foreignKeys/class-use/ForeignKey.html]

Uses of Interface foreignKeys.ForeignKey


|  |  |  |  |  |  |  |  |  |  |  |
| --- | --- | --- | --- | --- | --- | --- | --- | --- | --- | --- |
| |  |  |  |  |  |  |  |  | | --- | --- | --- | --- | --- | --- | --- | --- | | **Overview** | **Package** | **Class** | **Use** | **Tree** | **Deprecated** | **Index** | **Help** | | |  |
| PREV   NEXT | **FRAMES**    **NO FRAMES**     **All Classes** |


---


## **Uses of Interface foreignKeys.ForeignKey**

| Packages that use ForeignKey | |
| --- | --- |
| **foreignKeys** |  |
| **main** |  |

| Uses of ForeignKey in foreignKeys | |
| --- | --- |

| Classes in foreignKeys that implement ForeignKey | |
| --- | --- |
| `class` | `ForeignAESBlowfishKey` |
| `class` | `ForeignRSAKey` |

| Uses of ForeignKey in main | |
| --- | --- |

| Methods in main that return ForeignKey | |
| --- | --- |
| `ForeignKey` | `KeyManager.find(java.lang.String name, java.lang.String type, java.lang.String date)`             Finds a key in the keylist |
| `ForeignKey` | `User.getKey(java.lang.String name2, java.lang.String type, java.lang.String date)` |
| `ForeignKey` | `DNACrypt.getKey(java.lang.String name, java.lang.String type, java.lang.String date)` |

| Methods in main that return types with arguments of type ForeignKey | |
| --- | --- |
| `java.util.ArrayList<ForeignKey>` | `User.getKeyListe()` |
| `java.util.ArrayList<ForeignKey>` | `KeyManager.getKeyListe()` |
| `java.util.ArrayList<ForeignKey>` | `DNACrypt.getKeyListe()` |

| Methods in main with parameters of type ForeignKey | |
| --- | --- |
| `char[]` | `DNACrypt.binaryCrypt(byte[] file, ForeignKey key, int correction)`             Encrypts the inputfile by using the binary encryption |
| `byte[]` | `DNACrypt.binaryDecrypt(char[] genome, ForeignKey key, boolean inGenome, int correction)`             Decrypts a genome by using the binary decryption |
| `void` | `KeyManager.deleteKey(ForeignKey foreignKey)`             Deletes a key |
| `void` | `User.newKey(ForeignKey foreignKey)`             Adds a key to the KeyManager |
| `void` | `KeyManager.newKey(ForeignKey foreignKey)`             Adds a key to the keylist |

---


|  |  |  |  |  |  |  |  |  |  |  |
| --- | --- | --- | --- | --- | --- | --- | --- | --- | --- | --- |
| |  |  |  |  |  |  |  |  | | --- | --- | --- | --- | --- | --- | --- | --- | | **Overview** | **Package** | **Class** | **Use** | **Tree** | **Deprecated** | **Index** | **Help** | | |  |
| PREV   NEXT | **FRAMES**    **NO FRAMES**     **All Classes** |


---
