# Supplementary material for: DNA-based watermarks using the DNA-Crypt algorithm
Source: BMC Bioinformatics. 2007 May 29;8:176. doi: 10.1186/1471-2105-8-176 (PMC1904243; doi:10.1186/1471-2105-8-176)
Supplement: Additional file 1 — The DNA-Crypt v.2. [file 1471-2105-8-176-S1.zip › help/doc/foreignKeys/ForeignKey.html]

ForeignKey


|  |  |  |  |  |  |  |  |  |  |  |
| --- | --- | --- | --- | --- | --- | --- | --- | --- | --- | --- |
| |  |  |  |  |  |  |  |  | | --- | --- | --- | --- | --- | --- | --- | --- | | **Overview** | **Package** | **Class** | **Use** | **Tree** | **Deprecated** | **Index** | **Help** | | |  |
| **PREV CLASS**   **NEXT CLASS** | **FRAMES**    **NO FRAMES**     **All Classes** |
| SUMMARY: NESTED | FIELD | CONSTR | METHOD | DETAIL: FIELD | CONSTR | METHOD |


---


## foreignKeys Interface ForeignKey

**All Superinterfaces:**: java.io.Serializable

**All Known Implementing Classes:**: ForeignAESBlowfishKey, ForeignRSAKey

---

``` public interface ForeignKey extends java.io.Serializable ```

**Author:**
:   Dominik Heider

---

| **Method Summary** | |
| --- | --- |
| `java.util.Date` | `getDate()` |
| `java.lang.String` | `getName()` |
| `java.lang.String` | `getType()` |

| **Method Detail** |
| --- |

### getName

```
java.lang.String getName()
```

:   **Returns:**: the name of the owner

---


### getType

```
java.lang.String getType()
```

:   **Returns:**: the type of the key

---


### getDate

```
java.util.Date getDate()
```

:   **Returns:**: the time of creation


---


|  |  |  |  |  |  |  |  |  |  |  |
| --- | --- | --- | --- | --- | --- | --- | --- | --- | --- | --- |
| |  |  |  |  |  |  |  |  | | --- | --- | --- | --- | --- | --- | --- | --- | | **Overview** | **Package** | **Class** | **Use** | **Tree** | **Deprecated** | **Index** | **Help** | | |  |
| **PREV CLASS**   **NEXT CLASS** | **FRAMES**    **NO FRAMES**     **All Classes** |
| SUMMARY: NESTED | FIELD | CONSTR | METHOD | DETAIL: FIELD | CONSTR | METHOD |


---
