# Supplementary material for: DNA-based watermarks using the DNA-Crypt algorithm
Source: BMC Bioinformatics. 2007 May 29;8:176. doi: 10.1186/1471-2105-8-176 (PMC1904243; doi:10.1186/1471-2105-8-176)
Supplement: Additional file 1 — The DNA-Crypt v.2. [file 1471-2105-8-176-S1.zip › help/doc/foreignKeys/ForeignRSAKey.html]

ForeignRSAKey


|  |  |  |  |  |  |  |  |  |  |  |
| --- | --- | --- | --- | --- | --- | --- | --- | --- | --- | --- |
| |  |  |  |  |  |  |  |  | | --- | --- | --- | --- | --- | --- | --- | --- | | **Overview** | **Package** | **Class** | **Use** | **Tree** | **Deprecated** | **Index** | **Help** | | |  |
| **PREV CLASS**   NEXT CLASS | **FRAMES**    **NO FRAMES**     **All Classes** |
| SUMMARY: NESTED | FIELD | CONSTR | METHOD | DETAIL: FIELD | CONSTR | METHOD |


---


## foreignKeys Class ForeignRSAKey

```
java.lang.Object
  foreignKeys.ForeignRSAKey
```

**All Implemented Interfaces:**: ForeignKey, java.io.Serializable

---

``` public class ForeignRSAKey extends java.lang.Object implements ForeignKey, java.io.Serializable ```

**Author:**
:   Dominik

**See Also:**: Serialized Form

---

| **Constructor Summary** | |
| --- | --- |
| `ForeignRSAKey(java.lang.String name, java.lang.String type, java.security.Key key)` |


| **Method Summary** | |
| --- | --- |
| `byte[]` | `decrypt(byte[] code, javax.crypto.Cipher cipher)`             Decrypts a byte array using the Private Key |
| `byte[]` | `encrypt(byte[] text, javax.crypto.Cipher cipher)`             Encrypts a byte array using the Public Key |
| `java.util.Date` | `getDate()` |
| `java.lang.String` | `getName()` |
| `java.lang.String` | `getType()` |

| **Methods inherited from class java.lang.Object** |
| --- |
| `equals, getClass, hashCode, notify, notifyAll, toString, wait, wait, wait` |

| **Constructor Detail** |
| --- |

### ForeignRSAKey

```
public ForeignRSAKey(java.lang.String name,
                     java.lang.String type,
                     java.security.Key key)
```

**Parameters:**: `name` - of the the owner: `type` - of the key, RSA-Public or RSA-Private: `date` - of creation: `skeySpec` - the Secret Key


| **Method Detail** |
| --- |

### encrypt

```
public byte[] encrypt(byte[] text,
                      javax.crypto.Cipher cipher)
               throws java.security.GeneralSecurityException
```

:   Encrypts a byte array using the Public Key

    :   **Parameters:**: `text` - the byte array to encrypt: `cipher` - used for encryption **Returns:**: the encrypted byte array **Throws:**: `java.security.GeneralSecurityException`

---


### decrypt

```
public byte[] decrypt(byte[] code,
                      javax.crypto.Cipher cipher)
               throws java.security.GeneralSecurityException
```

:   Decrypts a byte array using the Private Key

    :   **Parameters:**: `code` - the byte array to decrypt: `cipher` - used for decryption **Returns:**: the decrypted byte array **Throws:**: `java.security.GeneralSecurityException`

---


### getName

```
public java.lang.String getName()
```

:   **Specified by:**: `getName` in interface `ForeignKey`
:   **Returns:**: Returns the name.

---


### getType

```
public java.lang.String getType()
```

:   **Specified by:**: `getType` in interface `ForeignKey`
:   **Returns:**: Returns the type.

---


### getDate

```
public java.util.Date getDate()
```

:   **Specified by:**: `getDate` in interface `ForeignKey`
:   **Returns:**: Returns the date.


---


|  |  |  |  |  |  |  |  |  |  |  |
| --- | --- | --- | --- | --- | --- | --- | --- | --- | --- | --- |
| |  |  |  |  |  |  |  |  | | --- | --- | --- | --- | --- | --- | --- | --- | | **Overview** | **Package** | **Class** | **Use** | **Tree** | **Deprecated** | **Index** | **Help** | | |  |
| **PREV CLASS**   NEXT CLASS | **FRAMES**    **NO FRAMES**     **All Classes** |
| SUMMARY: NESTED | FIELD | CONSTR | METHOD | DETAIL: FIELD | CONSTR | METHOD |


---
