# Supplementary material for: DNA-based watermarks using the DNA-Crypt algorithm
Source: BMC Bioinformatics. 2007 May 29;8:176. doi: 10.1186/1471-2105-8-176 (PMC1904243; doi:10.1186/1471-2105-8-176)
Supplement: Additional file 1 — The DNA-Crypt v.2. [file 1471-2105-8-176-S1.zip › help/doc/foreignKeys/package-frame.html]

foreignKeys


foreignKeys

|  |
| --- |
| Interfaces    *ForeignKey* |

|  |
| --- |
| Classes    ForeignAESBlowfishKey   ForeignRSAKey |
