# Supplementary material for: DNA-based watermarks using the DNA-Crypt algorithm
Source: BMC Bioinformatics. 2007 May 29;8:176. doi: 10.1186/1471-2105-8-176 (PMC1904243; doi:10.1186/1471-2105-8-176)
Supplement: Additional file 1 — The DNA-Crypt v.2. [file 1471-2105-8-176-S1.zip › help/doc/foreignKeys/package-tree.html]

foreignKeys Class Hierarchy


|  |  |  |  |  |  |  |  |  |  |  |
| --- | --- | --- | --- | --- | --- | --- | --- | --- | --- | --- |
| |  |  |  |  |  |  |  |  | | --- | --- | --- | --- | --- | --- | --- | --- | | **Overview** | **Package** | Class | Use | **Tree** | **Deprecated** | **Index** | **Help** | | |  |
| **PREV**   **NEXT** | **FRAMES**    **NO FRAMES**     **All Classes** |


---


## Hierarchy For Package foreignKeys

**Package Hierarchies:**: All Packages

---

## Class Hierarchy

- java.lang.Object
  - foreignKeys.**ForeignAESBlowfishKey** (implements foreignKeys.ForeignKey, java.io.Serializable)- foreignKeys.**ForeignRSAKey** (implements foreignKeys.ForeignKey, java.io.Serializable)

## Interface Hierarchy

- java.io.Serializable
  - foreignKeys.**ForeignKey**

---


|  |  |  |  |  |  |  |  |  |  |  |
| --- | --- | --- | --- | --- | --- | --- | --- | --- | --- | --- |
| |  |  |  |  |  |  |  |  | | --- | --- | --- | --- | --- | --- | --- | --- | | **Overview** | **Package** | Class | Use | **Tree** | **Deprecated** | **Index** | **Help** | | |  |
| **PREV**   **NEXT** | **FRAMES**    **NO FRAMES**     **All Classes** |


---
