# Supplementary material for: DNA-based watermarks using the DNA-Crypt algorithm
Source: BMC Bioinformatics. 2007 May 29;8:176. doi: 10.1186/1471-2105-8-176 (PMC1904243; doi:10.1186/1471-2105-8-176)
Supplement: Additional file 1 — The DNA-Crypt v.2. [file 1471-2105-8-176-S1.zip › help/doc/genome/Analyser.html]

Analyser


|  |  |  |  |  |  |  |  |  |  |  |
| --- | --- | --- | --- | --- | --- | --- | --- | --- | --- | --- |
| |  |  |  |  |  |  |  |  | | --- | --- | --- | --- | --- | --- | --- | --- | | **Overview** | **Package** | **Class** | **Use** | **Tree** | **Deprecated** | **Index** | **Help** | | |  |
| PREV CLASS   **NEXT CLASS** | **FRAMES**    **NO FRAMES**     **All Classes** |
| SUMMARY: NESTED | FIELD | CONSTR | METHOD | DETAIL: FIELD | CONSTR | METHOD |


---


## genome Class Analyser

```
java.lang.Object
  genome.Analyser
```

---

``` public class Analyser extends java.lang.Object ```

**Author:**
:   Dominik Heider

---

| **Field Summary** | |
| --- | --- |
| `int` | `Alanin` |
| `int` | `Arginin` |
| `int` | `Arginin2` |
| `int` | `Asparagin` |
| `int` | `Asparaginsaure` |
| `int` | `Cystein` |
| `int` | `gc` |
| `int` | `Glutamin` |
| `int` | `Glutaminsaure` |
| `int` | `Glycin` |
| `int` | `Histidin` |
| `int` | `Isoleucin` |
| `int` | `Leucin` |
| `int` | `Leucin2` |
| `int` | `Lysin` |
| `int` | `Methionin` |
| `int` | `Phenylalanin` |
| `int` | `Prolin` |
| `int` | `Serin` |
| `int` | `Serin2` |
| `int` | `Stopcodon` |
| `int` | `Threonin` |
| `int` | `Tryptophan` |
| `int` | `Tyrosin` |
| `int` | `Valin` |


| **Constructor Summary** | |
| --- | --- |
| `Analyser()`             Sets all Register to zero |


| **Method Summary** | |
| --- | --- |
| `void` | `analyse(char[] genome)`             analyses a given RNA sequence |
| `float` | `countAllAminoacids()` |
| `int` | `getAlanin()` |
| `int` | `getArginin()` |
| `int` | `getArginin2()` |
| `int` | `getAsparagin()` |
| `int` | `getAsparaginsaure()` |
| `int` | `getCystein()` |
| `int` | `getGlutamin()` |
| `int` | `getGlutaminsaure()` |
| `int` | `getGlycin()` |
| `int` | `getHistidin()` |
| `int` | `getIsoleucin()` |
| `int` | `getLeucin()` |
| `int` | `getLeucin2()` |
| `int` | `getLysin()` |
| `int` | `getMethionin()` |
| `int` | `getPhenylalanin()` |
| `int` | `getProlin()` |
| `int` | `getSerin()` |
| `int` | `getSerin2()` |
| `int` | `getStopcodon()` |
| `int` | `getThreonin()` |
| `int` | `getTryptophan()` |
| `int` | `getTyrosin()` |
| `int` | `getValin()` |
| `float` | `getVariables()` |

| **Methods inherited from class java.lang.Object** |
| --- |
| `equals, getClass, hashCode, notify, notifyAll, toString, wait, wait, wait` |

| **Field Detail** |
| --- |

### Alanin

```
public int Alanin
```

---


### Arginin

```
public int Arginin
```

---


### Arginin2

```
public int Arginin2
```

---


### Asparagin

```
public int Asparagin
```

---


### Asparaginsaure

```
public int Asparaginsaure
```

---


### Cystein

```
public int Cystein
```

---


### Glutamin

```
public int Glutamin
```

---


### Glutaminsaure

```
public int Glutaminsaure
```

---


### Glycin

```
public int Glycin
```

---


### Histidin

```
public int Histidin
```

---


### Isoleucin

```
public int Isoleucin
```

---


### Leucin

```
public int Leucin
```

---


### Leucin2

```
public int Leucin2
```

---


### Lysin

```
public int Lysin
```

---


### Methionin

```
public int Methionin
```

---


### Phenylalanin

```
public int Phenylalanin
```

---


### Prolin

```
public int Prolin
```

---


### Serin

```
public int Serin
```

---


### Serin2

```
public int Serin2
```

---


### Threonin

```
public int Threonin
```

---


### Tryptophan

```
public int Tryptophan
```

---


### Tyrosin

```
public int Tyrosin
```

---


### Valin

```
public int Valin
```

---


### Stopcodon

```
public int Stopcodon
```

---


### gc

```
public int gc
```


| **Constructor Detail** |
| --- |

### Analyser

```
public Analyser()
```

:   Sets all Register to zero


| **Method Detail** |
| --- |

### analyse

```
public void analyse(char[] genome)
```

:   analyses a given RNA sequence

    :   **Parameters:**: `genome` - the RNA sequence

---


### countAllAminoacids

```
public float countAllAminoacids()
```

:   **Returns:**: the count of all resulting aminoacids

---


### getVariables

```
public float getVariables()
```

:   **Returns:**: the count of the AminoAcids with synonymous substitutions

---


### getAlanin

```
public int getAlanin()
```

:   **Returns:**: Returns the count of Alanine.

---


### getArginin

```
public int getArginin()
```

:   **Returns:**: Returns the count of Arginine.

---


### getArginin2

```
public int getArginin2()
```

:   **Returns:**: Returns the count of the 2nd form of Arginine.

---


### getAsparagin

```
public int getAsparagin()
```

:   **Returns:**: Returns the count of Asparagine.

---


### getAsparaginsaure

```
public int getAsparaginsaure()
```

:   **Returns:**: Returns the count of Aspartic acid.

---


### getCystein

```
public int getCystein()
```

:   **Returns:**: Returns the count of Cysteine.

---


### getGlutamin

```
public int getGlutamin()
```

:   **Returns:**: Returns the count of Glutamine.

---


### getGlutaminsaure

```
public int getGlutaminsaure()
```

:   **Returns:**: Returns the count of Glutamic acid.

---


### getGlycin

```
public int getGlycin()
```

:   **Returns:**: Returns the count of Glycine.

---


### getHistidin

```
public int getHistidin()
```

:   **Returns:**: Returns the count of Histidine.

---


### getIsoleucin

```
public int getIsoleucin()
```

:   **Returns:**: Returns the count of Isoleucine.

---


### getLeucin

```
public int getLeucin()
```

:   **Returns:**: Returns the count of Leucine.

---


### getLeucin2

```
public int getLeucin2()
```

:   **Returns:**: Returns the count of the 2nd form of Leucine.

---


### getLysin

```
public int getLysin()
```

:   **Returns:**: Returns the count of Lysine.

---


### getMethionin

```
public int getMethionin()
```

:   **Returns:**: Returns the count of Methionine.

---


### getPhenylalanin

```
public int getPhenylalanin()
```

:   **Returns:**: Returns the count of Phenylalanine.

---


### getProlin

```
public int getProlin()
```

:   **Returns:**: Returns the count of Proline.

---


### getSerin

```
public int getSerin()
```

:   **Returns:**: Returns the count of Serine.

---


### getSerin2

```
public int getSerin2()
```

:   **Returns:**: Returns the count of the 2nd form of Serine.

---


### getStopcodon

```
public int getStopcodon()
```

:   **Returns:**: Returns the count of stopcodon.

---


### getThreonin

```
public int getThreonin()
```

:   **Returns:**: Returns the count of Threonine.

---


### getTryptophan

```
public int getTryptophan()
```

:   **Returns:**: Returns the count of Tryptophan.

---


### getTyrosin

```
public int getTyrosin()
```

:   **Returns:**: Returns the count of Tyrosine.

---


### getValin

```
public int getValin()
```

:   **Returns:**: Returns the count of Valine.


---


|  |  |  |  |  |  |  |  |  |  |  |
| --- | --- | --- | --- | --- | --- | --- | --- | --- | --- | --- |
| |  |  |  |  |  |  |  |  | | --- | --- | --- | --- | --- | --- | --- | --- | | **Overview** | **Package** | **Class** | **Use** | **Tree** | **Deprecated** | **Index** | **Help** | | |  |
| PREV CLASS   **NEXT CLASS** | **FRAMES**    **NO FRAMES**     **All Classes** |
| SUMMARY: NESTED | FIELD | CONSTR | METHOD | DETAIL: FIELD | CONSTR | METHOD |


---
