# Supplementary material for: DNA-based watermarks using the DNA-Crypt algorithm
Source: BMC Bioinformatics. 2007 May 29;8:176. doi: 10.1186/1471-2105-8-176 (PMC1904243; doi:10.1186/1471-2105-8-176)
Supplement: Additional file 1 — The DNA-Crypt v.2. [file 1471-2105-8-176-S1.zip › help/doc/genome/GenomeOperator.html]

GenomeOperator


|  |  |  |  |  |  |  |  |  |  |  |
| --- | --- | --- | --- | --- | --- | --- | --- | --- | --- | --- |
| |  |  |  |  |  |  |  |  | | --- | --- | --- | --- | --- | --- | --- | --- | | **Overview** | **Package** | **Class** | **Use** | **Tree** | **Deprecated** | **Index** | **Help** | | |  |
| **PREV CLASS**   NEXT CLASS | **FRAMES**    **NO FRAMES**     **All Classes** |
| SUMMARY: NESTED | FIELD | CONSTR | METHOD | DETAIL: FIELD | CONSTR | METHOD |


---


## genome Class GenomeOperator

```
java.lang.Object
  genome.GenomeOperator
```

---

``` public class GenomeOperator extends java.lang.Object ```

GenomeOperator transcribes DNA sequences to RNA, translates
RNA to proteine sequences or reverse transcribes RNA to DNA.

**Author:**
:   Dominik Heider

---

| **Constructor Summary** | |
| --- | --- |
| `GenomeOperator()` |


| **Method Summary** | |
| --- | --- |
| `char[]` | `reverse(char[] genome)`             reverse transcriptase a RNA sequence |
| `char[]` | `transkribe(char[] genome)`             transcribes a given DNA sequence |
| `char[]` | `translate(char[] genome)`             translated a given RNA sequence |

| **Methods inherited from class java.lang.Object** |
| --- |
| `equals, getClass, hashCode, notify, notifyAll, toString, wait, wait, wait` |

| **Constructor Detail** |
| --- |

### GenomeOperator

```
public GenomeOperator()
```


| **Method Detail** |
| --- |

### transkribe

```
public char[] transkribe(char[] genome)
```

:   transcribes a given DNA sequence

    :   **Parameters:**: `genome` - the DNA sequence **Returns:**: the RNA sequence

---


### translate

```
public char[] translate(char[] genome)
```

:   translated a given RNA sequence

    :   **Parameters:**: `genome` - the RNA sequence **Returns:**: the proteine sequence

---


### reverse

```
public char[] reverse(char[] genome)
```

:   reverse transcriptase a RNA sequence

    :   **Parameters:**: `genome` - the RNA sequence **Returns:**: the DNA sequence


---


|  |  |  |  |  |  |  |  |  |  |  |
| --- | --- | --- | --- | --- | --- | --- | --- | --- | --- | --- |
| |  |  |  |  |  |  |  |  | | --- | --- | --- | --- | --- | --- | --- | --- | | **Overview** | **Package** | **Class** | **Use** | **Tree** | **Deprecated** | **Index** | **Help** | | |  |
| **PREV CLASS**   NEXT CLASS | **FRAMES**    **NO FRAMES**     **All Classes** |
| SUMMARY: NESTED | FIELD | CONSTR | METHOD | DETAIL: FIELD | CONSTR | METHOD |


---
