# Supplementary material for: DNA-based watermarks using the DNA-Crypt algorithm
Source: BMC Bioinformatics. 2007 May 29;8:176. doi: 10.1186/1471-2105-8-176 (PMC1904243; doi:10.1186/1471-2105-8-176)
Supplement: Additional file 1 — The DNA-Crypt v.2. [file 1471-2105-8-176-S1.zip › help/doc/genome/package-summary.html]

genome


|  |  |  |  |  |  |  |  |  |  |  |
| --- | --- | --- | --- | --- | --- | --- | --- | --- | --- | --- |
| |  |  |  |  |  |  |  |  | | --- | --- | --- | --- | --- | --- | --- | --- | | **Overview** | **Package** | Class | **Use** | **Tree** | **Deprecated** | **Index** | **Help** | | |  |
| **PREV PACKAGE**   **NEXT PACKAGE** | **FRAMES**    **NO FRAMES**     **All Classes** |


---

## Package genome

| **Class Summary** | |
| --- | --- |
| **Analyser** |  |
| **GenomeOperator** | GenomeOperator transcribes DNA sequences to RNA, translates RNA to proteine sequences or reverse transcribes RNA to DNA. |

---


|  |  |  |  |  |  |  |  |  |  |  |
| --- | --- | --- | --- | --- | --- | --- | --- | --- | --- | --- |
| |  |  |  |  |  |  |  |  | | --- | --- | --- | --- | --- | --- | --- | --- | | **Overview** | **Package** | Class | **Use** | **Tree** | **Deprecated** | **Index** | **Help** | | |  |
| **PREV PACKAGE**   **NEXT PACKAGE** | **FRAMES**    **NO FRAMES**     **All Classes** |


---
