# Supplementary material for: DNA-based watermarks using the DNA-Crypt algorithm
Source: BMC Bioinformatics. 2007 May 29;8:176. doi: 10.1186/1471-2105-8-176 (PMC1904243; doi:10.1186/1471-2105-8-176)
Supplement: Additional file 1 — The DNA-Crypt v.2. [file 1471-2105-8-176-S1.zip › help/doc/index-files/index-1.html]

A-Index


|  |  |  |  |  |  |  |  |  |  |  |
| --- | --- | --- | --- | --- | --- | --- | --- | --- | --- | --- |
| |  |  |  |  |  |  |  |  | | --- | --- | --- | --- | --- | --- | --- | --- | | **Overview** | Package | Class | Use | **Tree** | **Deprecated** | **Index** | **Help** | | |  |
| PREV LETTER   **NEXT LETTER** | **FRAMES**    **NO FRAMES**     **All Classes** |


A B C D E F G H I K L M N O P R S T U V W 

---


## **A**

**AES** - Class in symmetric: **AES()** - Constructor for class symmetric.AES: creates a secret key for the AES algorithm. **Alanin** - Variable in class genome.Analyser: **AminoSteg** - Class in steg: **AminoSteg(DNACrypt)** - Constructor for class steg.AminoSteg: Creates an instance of AminoSteg **analyse(char[])** - Method in class genome.Analyser: analyses a given RNA sequence **Analyser** - Class in genome: **Analyser()** - Constructor for class genome.Analyser: Sets all Register to zero **Arginin** - Variable in class genome.Analyser: **Arginin2** - Variable in class genome.Analyser: **Asparagin** - Variable in class genome.Analyser: **Asparaginsaure** - Variable in class genome.Analyser: **asymmetric** - package asymmetric

---


|  |  |  |  |  |  |  |  |  |  |  |
| --- | --- | --- | --- | --- | --- | --- | --- | --- | --- | --- |
| |  |  |  |  |  |  |  |  | | --- | --- | --- | --- | --- | --- | --- | --- | | **Overview** | Package | Class | Use | **Tree** | **Deprecated** | **Index** | **Help** | | |  |
| PREV LETTER   **NEXT LETTER** | **FRAMES**    **NO FRAMES**     **All Classes** |


A B C D E F G H I K L M N O P R S T U V W 

---
