# Supplementary material for: DNA-based watermarks using the DNA-Crypt algorithm
Source: BMC Bioinformatics. 2007 May 29;8:176. doi: 10.1186/1471-2105-8-176 (PMC1904243; doi:10.1186/1471-2105-8-176)
Supplement: Additional file 1 — The DNA-Crypt v.2. [file 1471-2105-8-176-S1.zip › help/doc/index-files/index-11.html]

L-Index


|  |  |  |  |  |  |  |  |  |  |  |
| --- | --- | --- | --- | --- | --- | --- | --- | --- | --- | --- |
| |  |  |  |  |  |  |  |  | | --- | --- | --- | --- | --- | --- | --- | --- | | **Overview** | Package | Class | Use | **Tree** | **Deprecated** | **Index** | **Help** | | |  |
| **PREV LETTER**   **NEXT LETTER** | **FRAMES**    **NO FRAMES**     **All Classes** |


A B C D E F G H I K L M N O P R S T U V W 

---


## **L**

**Leucin** - Variable in class genome.Analyser: **Leucin2** - Variable in class genome.Analyser: **loadFile(File)** - Method in class main.DNACrypt: Loads a file **loadGenome(File)** - Method in class main.DNACrypt: Loads a genome **loadOneTimePad(File)** - Method in class main.DNACrypt: Loads a One Time Pad **loadProperties()** - Method in class main.DNACrypt: Loads the Property file **loadUserVerwaltung()** - Method in class main.DNACrypt: Loads the UserManager **loginUser(User)** - Method in class main.DNACrypt: Sets the current User to the given one **logoutUser()** - Method in class main.DNACrypt: Logs out the current User **Lysin** - Variable in class genome.Analyser

---


|  |  |  |  |  |  |  |  |  |  |  |
| --- | --- | --- | --- | --- | --- | --- | --- | --- | --- | --- |
| |  |  |  |  |  |  |  |  | | --- | --- | --- | --- | --- | --- | --- | --- | | **Overview** | Package | Class | Use | **Tree** | **Deprecated** | **Index** | **Help** | | |  |
| **PREV LETTER**   **NEXT LETTER** | **FRAMES**    **NO FRAMES**     **All Classes** |


A B C D E F G H I K L M N O P R S T U V W 

---
