# Supplementary material for: DNA-based watermarks using the DNA-Crypt algorithm
Source: BMC Bioinformatics. 2007 May 29;8:176. doi: 10.1186/1471-2105-8-176 (PMC1904243; doi:10.1186/1471-2105-8-176)
Supplement: Additional file 1 — The DNA-Crypt v.2. [file 1471-2105-8-176-S1.zip › help/doc/index-files/index-17.html]

S-Index


|  |  |  |  |  |  |  |  |  |  |  |
| --- | --- | --- | --- | --- | --- | --- | --- | --- | --- | --- |
| |  |  |  |  |  |  |  |  | | --- | --- | --- | --- | --- | --- | --- | --- | | **Overview** | Package | Class | Use | **Tree** | **Deprecated** | **Index** | **Help** | | |  |
| **PREV LETTER**   **NEXT LETTER** | **FRAMES**    **NO FRAMES**     **All Classes** |


A B C D E F G H I K L M N O P R S T U V W 

---


## **S**

**saveFile(String, Object)** - Method in class main.DNACrypt: Saves a file **saveOutput(File, String)** - Method in class main.DNACrypt: Saves the output to file system **saveOutput(File)** - Method in class main.DNACrypt: Saves the output to file system **Serin** - Variable in class genome.Analyser: **Serin2** - Variable in class genome.Analyser: **setBinaryFlag(boolean)** - Method in class main.DNACrypt: **setGenome(char[])** - Method in class main.DNACrypt: **setInputfile(byte[])** - Method in class main.DNACrypt: **setLogin(String)** - Method in class main.User: **setName(String)** - Method in class main.User: **setOutputfile(byte[])** - Method in class main.DNACrypt: **setPasswort(String)** - Method in class main.User: **setUser(String, String, String)** - Method in class main.DNACrypt: Sets the attributes of the current User **setVorname(String)** - Method in class main.User: **steg** - package steg: **Stopcodon** - Variable in class genome.Analyser: **stretch(char[], char[])** - Method in class steg.AminoSteg: hides a RNA sequence in a genome (RNA). **symmetric** - package symmetric

---


|  |  |  |  |  |  |  |  |  |  |  |
| --- | --- | --- | --- | --- | --- | --- | --- | --- | --- | --- |
| |  |  |  |  |  |  |  |  | | --- | --- | --- | --- | --- | --- | --- | --- | | **Overview** | Package | Class | Use | **Tree** | **Deprecated** | **Index** | **Help** | | |  |
| **PREV LETTER**   **NEXT LETTER** | **FRAMES**    **NO FRAMES**     **All Classes** |


A B C D E F G H I K L M N O P R S T U V W 

---
