# Supplementary material for: DNA-based watermarks using the DNA-Crypt algorithm
Source: BMC Bioinformatics. 2007 May 29;8:176. doi: 10.1186/1471-2105-8-176 (PMC1904243; doi:10.1186/1471-2105-8-176)
Supplement: Additional file 1 — The DNA-Crypt v.2. [file 1471-2105-8-176-S1.zip › help/doc/index-files/index-2.html]

B-Index


|  |  |  |  |  |  |  |  |  |  |  |
| --- | --- | --- | --- | --- | --- | --- | --- | --- | --- | --- |
| |  |  |  |  |  |  |  |  | | --- | --- | --- | --- | --- | --- | --- | --- | | **Overview** | Package | Class | Use | **Tree** | **Deprecated** | **Index** | **Help** | | |  |
| **PREV LETTER**   **NEXT LETTER** | **FRAMES**    **NO FRAMES**     **All Classes** |


A B C D E F G H I K L M N O P R S T U V W 

---


## **B**

**binaryCrypt(byte[], ForeignKey, int)** - Method in class main.DNACrypt: Encrypts the inputfile by using the binary encryption **binaryDecrypt(char[], ForeignKey, boolean, int)** - Method in class main.DNACrypt: Decrypts a genome by using the binary decryption **BitCoding** - Class in steg: **BitCoding(DNACrypt)** - Constructor for class steg.BitCoding: Creates an instance of BitCoding **Blowfish** - Class in symmetric: **Blowfish()** - Constructor for class symmetric.Blowfish: creates a secret key for the Blowfish algorithm. **BrowserControl** - Class in main: **BrowserControl()** - Constructor for class main.BrowserControl

---


|  |  |  |  |  |  |  |  |  |  |  |
| --- | --- | --- | --- | --- | --- | --- | --- | --- | --- | --- |
| |  |  |  |  |  |  |  |  | | --- | --- | --- | --- | --- | --- | --- | --- | | **Overview** | Package | Class | Use | **Tree** | **Deprecated** | **Index** | **Help** | | |  |
| **PREV LETTER**   **NEXT LETTER** | **FRAMES**    **NO FRAMES**     **All Classes** |


A B C D E F G H I K L M N O P R S T U V W 

---
