# Supplementary material for: DNA-based watermarks using the DNA-Crypt algorithm
Source: BMC Bioinformatics. 2007 May 29;8:176. doi: 10.1186/1471-2105-8-176 (PMC1904243; doi:10.1186/1471-2105-8-176)
Supplement: Additional file 1 — The DNA-Crypt v.2. [file 1471-2105-8-176-S1.zip › help/doc/index-files/index-4.html]

D-Index


|  |  |  |  |  |  |  |  |  |  |  |
| --- | --- | --- | --- | --- | --- | --- | --- | --- | --- | --- |
| |  |  |  |  |  |  |  |  | | --- | --- | --- | --- | --- | --- | --- | --- | | **Overview** | Package | Class | Use | **Tree** | **Deprecated** | **Index** | **Help** | | |  |
| **PREV LETTER**   **NEXT LETTER** | **FRAMES**    **NO FRAMES**     **All Classes** |


A B C D E F G H I K L M N O P R S T U V W 

---


## **D**

**decode(char[])** - Method in class steg.BitCoding: Filters a file out of a RNA sequence **decode(char[])** - Method in class steg.Clelland: Decodes a character array out of a RNA sequence **decode(byte[])** - Method in interface steg.CorrectionCode: Decodes a bytearray **decode(byte[])** - Method in class steg.HammingCode: Decodes a byte array **decode(byte)** - Method in class steg.HammingCode: Decodes a byte h7, h6, h5, h4, h3, h2, h1, h0 p = h7 ^ h6 ^ h5 ^ h4 ^ h3 ^ h2 ^ h1 ^ h0 c0 = h7 ^ h5 ^ h1 ^ h0 c1 = h7 ^ h3 ^ h2 ^ h1 c2 = h5 ^ h4 ^ h3 ^ h1 If the parity, p, is correct (equal to 1) then either 0 or 2 errors occurred. **decode(byte[])** - Method in class steg.NonCorrection: **decode(byte[])** - Method in class steg.WDHC: Decodes a byte array **decrypt(byte[], Cipher)** - Method in class foreignKeys.ForeignAESBlowfishKey: Decrypts a byte array using AES or Blowfish. **decrypt(byte[], Cipher)** - Method in class foreignKeys.ForeignRSAKey: Decrypts a byte array using the Private Key **defaultProperties()** - Method in class main.DNACrypt: Sets Default Properties **deleteKey(String, String, String)** - Method in class main.DNACrypt: Deletes a key **deleteKey(String, String, String)** - Method in class main.KeyManager: Deletes a key **deleteKey(ForeignKey)** - Method in class main.KeyManager: Deletes a key **deleteKey(String, String, String)** - Method in class main.User: Deletes a Key **deleteUser(String)** - Method in class main.UserManager: Deletes a User **destretch(char[])** - Method in class steg.AminoSteg: filters a sequence out of a given genome by reading the header. **displayURL(String)** - Static method in class main.BrowserControl: Display a file in the system browser. **DNACrypt** - Class in main: **DNACrypt()** - Constructor for class main.DNACrypt: Creates an instance of DNACrypt

---


|  |  |  |  |  |  |  |  |  |  |  |
| --- | --- | --- | --- | --- | --- | --- | --- | --- | --- | --- |
| |  |  |  |  |  |  |  |  | | --- | --- | --- | --- | --- | --- | --- | --- | | **Overview** | Package | Class | Use | **Tree** | **Deprecated** | **Index** | **Help** | | |  |
| **PREV LETTER**   **NEXT LETTER** | **FRAMES**    **NO FRAMES**     **All Classes** |


A B C D E F G H I K L M N O P R S T U V W 

---
