# Supplementary material for: DNA-based watermarks using the DNA-Crypt algorithm
Source: BMC Bioinformatics. 2007 May 29;8:176. doi: 10.1186/1471-2105-8-176 (PMC1904243; doi:10.1186/1471-2105-8-176)
Supplement: Additional file 1 — The DNA-Crypt v.2. [file 1471-2105-8-176-S1.zip › help/doc/index-files/index-5.html]

E-Index


|  |  |  |  |  |  |  |  |  |  |  |
| --- | --- | --- | --- | --- | --- | --- | --- | --- | --- | --- |
| |  |  |  |  |  |  |  |  | | --- | --- | --- | --- | --- | --- | --- | --- | | **Overview** | Package | Class | Use | **Tree** | **Deprecated** | **Index** | **Help** | | |  |
| **PREV LETTER**   **NEXT LETTER** | **FRAMES**    **NO FRAMES**     **All Classes** |


A B C D E F G H I K L M N O P R S T U V W 

---


## **E**

**encode(byte[])** - Method in class steg.BitCoding: Encodes a given byte array into an RNA sequence **encode(char[])** - Method in class steg.Clelland: Encodes a character array into a RNA sequence **encode(byte[])** - Method in interface steg.CorrectionCode: Encodes a bytearray **encode(byte[])** - Method in class steg.HammingCode: Encodes a byte array **encode(byte)** - Method in class steg.HammingCode: The first four bits are used. **encode(byte[])** - Method in class steg.NonCorrection: **encode(byte[])** - Method in class steg.WDHC: Encodes a byte array **encrypt(byte[], Cipher)** - Method in class foreignKeys.ForeignAESBlowfishKey: encrypts a byte array using AES or Blowfish. **encrypt(byte[], Cipher)** - Method in class foreignKeys.ForeignRSAKey: Encrypts a byte array using the Public Key **existUser(String)** - Method in class main.DNACrypt: **existUser(String)** - Method in class main.UserManager: **exit()** - Method in class main.DNACrypt: Exiting DNACrypt **exportKey(String, String, String, File)** - Method in class main.DNACrypt: Exports a key to the file system

---


|  |  |  |  |  |  |  |  |  |  |  |
| --- | --- | --- | --- | --- | --- | --- | --- | --- | --- | --- |
| |  |  |  |  |  |  |  |  | | --- | --- | --- | --- | --- | --- | --- | --- | | **Overview** | Package | Class | Use | **Tree** | **Deprecated** | **Index** | **Help** | | |  |
| **PREV LETTER**   **NEXT LETTER** | **FRAMES**    **NO FRAMES**     **All Classes** |


A B C D E F G H I K L M N O P R S T U V W 

---
