# Supplementary material for: DNA-based watermarks using the DNA-Crypt algorithm
Source: BMC Bioinformatics. 2007 May 29;8:176. doi: 10.1186/1471-2105-8-176 (PMC1904243; doi:10.1186/1471-2105-8-176)
Supplement: Additional file 1 — The DNA-Crypt v.2. [file 1471-2105-8-176-S1.zip › help/doc/index-files/index-6.html]

F-Index


|  |  |  |  |  |  |  |  |  |  |  |
| --- | --- | --- | --- | --- | --- | --- | --- | --- | --- | --- |
| |  |  |  |  |  |  |  |  | | --- | --- | --- | --- | --- | --- | --- | --- | | **Overview** | Package | Class | Use | **Tree** | **Deprecated** | **Index** | **Help** | | |  |
| **PREV LETTER**   **NEXT LETTER** | **FRAMES**    **NO FRAMES**     **All Classes** |


A B C D E F G H I K L M N O P R S T U V W 

---


## **F**

**find(String, String, String)** - Method in class main.KeyManager: Finds a key in the keylist **find(String)** - Method in class main.UserManager: Finds a User by login **findUser(String)** - Method in class main.DNACrypt: Finds a User by login **ForeignAESBlowfishKey** - Class in foreignKeys: **ForeignAESBlowfishKey(String, String, SecretKeySpec)** - Constructor for class foreignKeys.ForeignAESBlowfishKey: Creates a ForeignAESBlowfishKey **ForeignKey** - Interface in foreignKeys: **foreignKeys** - package foreignKeys: **ForeignRSAKey** - Class in foreignKeys: **ForeignRSAKey(String, String, Key)** - Constructor for class foreignKeys.ForeignRSAKey

---


|  |  |  |  |  |  |  |  |  |  |  |
| --- | --- | --- | --- | --- | --- | --- | --- | --- | --- | --- |
| |  |  |  |  |  |  |  |  | | --- | --- | --- | --- | --- | --- | --- | --- | | **Overview** | Package | Class | Use | **Tree** | **Deprecated** | **Index** | **Help** | | |  |
| **PREV LETTER**   **NEXT LETTER** | **FRAMES**    **NO FRAMES**     **All Classes** |


A B C D E F G H I K L M N O P R S T U V W 

---
