# Supplementary material for: DNA-based watermarks using the DNA-Crypt algorithm
Source: BMC Bioinformatics. 2007 May 29;8:176. doi: 10.1186/1471-2105-8-176 (PMC1904243; doi:10.1186/1471-2105-8-176)
Supplement: Additional file 1 — The DNA-Crypt v.2. [file 1471-2105-8-176-S1.zip › help/doc/index-files/index-7.html]

G-Index


|  |  |  |  |  |  |  |  |  |  |  |
| --- | --- | --- | --- | --- | --- | --- | --- | --- | --- | --- |
| |  |  |  |  |  |  |  |  | | --- | --- | --- | --- | --- | --- | --- | --- | | **Overview** | Package | Class | Use | **Tree** | **Deprecated** | **Index** | **Help** | | |  |
| **PREV LETTER**   **NEXT LETTER** | **FRAMES**    **NO FRAMES**     **All Classes** |


A B C D E F G H I K L M N O P R S T U V W 

---


## **G**

**gc** - Variable in class genome.Analyser: **genome** - package genome: **GenomeOperator** - Class in genome: GenomeOperator transcribes DNA sequences to RNA, translates RNA to proteine sequences or reverse transcribes RNA to DNA. **GenomeOperator()** - Constructor for class genome.GenomeOperator: **getAlanin()** - Method in class genome.Analyser: **getArginin()** - Method in class genome.Analyser: **getArginin2()** - Method in class genome.Analyser: **getAsparagin()** - Method in class genome.Analyser: **getAsparaginsaure()** - Method in class genome.Analyser: **getbinaryFlag()** - Method in class main.DNACrypt: **getCystein()** - Method in class genome.Analyser: **getDate()** - Method in class foreignKeys.ForeignAESBlowfishKey: **getDate()** - Method in interface foreignKeys.ForeignKey: **getDate()** - Method in class foreignKeys.ForeignRSAKey: **getGenome()** - Method in class main.DNACrypt: **getGlutamin()** - Method in class genome.Analyser: **getGlutaminsaure()** - Method in class genome.Analyser: **getGlycin()** - Method in class genome.Analyser: **getHistidin()** - Method in class genome.Analyser: **getInputfile()** - Method in class main.DNACrypt: **getIsoleucin()** - Method in class genome.Analyser: **getKey(String, String, String)** - Method in class main.DNACrypt: **getKey(String, String, String)** - Method in class main.User: **getKey()** - Method in class symmetric.OneTimePad: **getKeyListe()** - Method in class main.DNACrypt: **getKeyListe()** - Method in class main.KeyManager: **getKeyListe()** - Method in class main.User: **getKeymanager()** - Method in class main.User: **getLeucin()** - Method in class genome.Analyser: **getLeucin2()** - Method in class genome.Analyser: **getLogin()** - Method in class main.User: **getLysin()** - Method in class genome.Analyser: **getMethionin()** - Method in class genome.Analyser: **getName()** - Method in class foreignKeys.ForeignAESBlowfishKey: **getName()** - Method in interface foreignKeys.ForeignKey: **getName()** - Method in class foreignKeys.ForeignRSAKey: **getName()** - Method in class main.User: **getPasswort()** - Method in class main.User: **getPhenylalanin()** - Method in class genome.Analyser: **getPrivate()** - Method in class asymmetric.RSA: **getProlin()** - Method in class genome.Analyser: **getProperties()** - Method in class main.DNACrypt: **getPublic()** - Method in class asymmetric.RSA: **getSerin()** - Method in class genome.Analyser: **getSerin2()** - Method in class genome.Analyser: **getSkeySpec()** - Method in class symmetric.AES: **getSkeySpec()** - Method in class symmetric.Blowfish: **getStopcodon()** - Method in class genome.Analyser: **getThreonin()** - Method in class genome.Analyser: **getTryptophan()** - Method in class genome.Analyser: **getType()** - Method in class foreignKeys.ForeignAESBlowfishKey: **getType()** - Method in interface foreignKeys.ForeignKey: **getType()** - Method in class foreignKeys.ForeignRSAKey: **getTyrosin()** - Method in class genome.Analyser: **getUser()** - Method in class main.DNACrypt: **getValin()** - Method in class genome.Analyser: **getVariables()** - Method in class genome.Analyser: **getVorname()** - Method in class main.User: **Glutamin** - Variable in class genome.Analyser: **Glutaminsaure** - Variable in class genome.Analyser: **Glycin** - Variable in class genome.Analyser

---


|  |  |  |  |  |  |  |  |  |  |  |
| --- | --- | --- | --- | --- | --- | --- | --- | --- | --- | --- |
| |  |  |  |  |  |  |  |  | | --- | --- | --- | --- | --- | --- | --- | --- | | **Overview** | Package | Class | Use | **Tree** | **Deprecated** | **Index** | **Help** | | |  |
| **PREV LETTER**   **NEXT LETTER** | **FRAMES**    **NO FRAMES**     **All Classes** |


A B C D E F G H I K L M N O P R S T U V W 

---
