# Supplementary material for: DNA-based watermarks using the DNA-Crypt algorithm
Source: BMC Bioinformatics. 2007 May 29;8:176. doi: 10.1186/1471-2105-8-176 (PMC1904243; doi:10.1186/1471-2105-8-176)
Supplement: Additional file 1 — The DNA-Crypt v.2. [file 1471-2105-8-176-S1.zip › help/doc/main/BrowserControl.html]

BrowserControl


|  |  |  |  |  |  |  |  |  |  |  |
| --- | --- | --- | --- | --- | --- | --- | --- | --- | --- | --- |
| |  |  |  |  |  |  |  |  | | --- | --- | --- | --- | --- | --- | --- | --- | | **Overview** | **Package** | **Class** | **Use** | **Tree** | **Deprecated** | **Index** | **Help** | | |  |
| PREV CLASS   **NEXT CLASS** | **FRAMES**    **NO FRAMES**     **All Classes** |
| SUMMARY: NESTED | FIELD | CONSTR | METHOD | DETAIL: FIELD | CONSTR | METHOD |


---


## main Class BrowserControl

```
java.lang.Object
  main.BrowserControl
```

---

``` public class BrowserControl extends java.lang.Object ```

---

| **Constructor Summary** | |
| --- | --- |
| `BrowserControl()` |


| **Method Summary** | |
| --- | --- |
| `static void` | `displayURL(java.lang.String url)`             Display a file in the system browser. |
| `static boolean` | `isWindowsPlatform()`             Try to determine whether this application is running under Windows or some other platform by examing the "os.name" property. |

| **Methods inherited from class java.lang.Object** |
| --- |
| `equals, getClass, hashCode, notify, notifyAll, toString, wait, wait, wait` |

| **Constructor Detail** |
| --- |

### BrowserControl

```
public BrowserControl()
```


| **Method Detail** |
| --- |

### displayURL

```
public static void displayURL(java.lang.String url)
```

:   Display a file in the system browser. If you want to display a
    file, you must include the absolute path name.

    :   **Parameters:**: `url` - the file's url (the url must start with either "http://" or "file://").

---


### isWindowsPlatform

```
public static boolean isWindowsPlatform()
```

:   Try to determine whether this application is running under Windows
    or some other platform by examing the "os.name" property.

    :   **Returns:**: true if this application is running under a Windows OS


---


|  |  |  |  |  |  |  |  |  |  |  |
| --- | --- | --- | --- | --- | --- | --- | --- | --- | --- | --- |
| |  |  |  |  |  |  |  |  | | --- | --- | --- | --- | --- | --- | --- | --- | | **Overview** | **Package** | **Class** | **Use** | **Tree** | **Deprecated** | **Index** | **Help** | | |  |
| PREV CLASS   **NEXT CLASS** | **FRAMES**    **NO FRAMES**     **All Classes** |
| SUMMARY: NESTED | FIELD | CONSTR | METHOD | DETAIL: FIELD | CONSTR | METHOD |


---
