# Supplementary material for: DNA-based watermarks using the DNA-Crypt algorithm
Source: BMC Bioinformatics. 2007 May 29;8:176. doi: 10.1186/1471-2105-8-176 (PMC1904243; doi:10.1186/1471-2105-8-176)
Supplement: Additional file 1 — The DNA-Crypt v.2. [file 1471-2105-8-176-S1.zip › help/doc/main/class-use/User.html]

Uses of Class main.User


|  |  |  |  |  |  |  |  |  |  |  |
| --- | --- | --- | --- | --- | --- | --- | --- | --- | --- | --- |
| |  |  |  |  |  |  |  |  | | --- | --- | --- | --- | --- | --- | --- | --- | | **Overview** | **Package** | **Class** | **Use** | **Tree** | **Deprecated** | **Index** | **Help** | | |  |
| PREV   NEXT | **FRAMES**    **NO FRAMES**     **All Classes** |


---


## **Uses of Class main.User**

| Packages that use User | |
| --- | --- |
| **main** |  |

| Uses of User in main | |
| --- | --- |

| Methods in main that return User | |
| --- | --- |
| `User` | `UserManager.find(java.lang.String login)`             Finds a User by login |
| `User` | `DNACrypt.findUser(java.lang.String login)`             Finds a User by login |
| `User` | `DNACrypt.getUser()` |

| Methods in main with parameters of type User | |
| --- | --- |
| `void` | `DNACrypt.loginUser(User user)`             Sets the current User to the given one |
| `void` | `UserManager.newUser(User user)`             Adds a new User to the userlist |

---


|  |  |  |  |  |  |  |  |  |  |  |
| --- | --- | --- | --- | --- | --- | --- | --- | --- | --- | --- |
| |  |  |  |  |  |  |  |  | | --- | --- | --- | --- | --- | --- | --- | --- | | **Overview** | **Package** | **Class** | **Use** | **Tree** | **Deprecated** | **Index** | **Help** | | |  |
| PREV   NEXT | **FRAMES**    **NO FRAMES**     **All Classes** |


---
