# Supplementary material for: DNA-based watermarks using the DNA-Crypt algorithm
Source: BMC Bioinformatics. 2007 May 29;8:176. doi: 10.1186/1471-2105-8-176 (PMC1904243; doi:10.1186/1471-2105-8-176)
Supplement: Additional file 1 — The DNA-Crypt v.2. [file 1471-2105-8-176-S1.zip › help/doc/main/DNACrypt.html]

DNACrypt


|  |  |  |  |  |  |  |  |  |  |  |
| --- | --- | --- | --- | --- | --- | --- | --- | --- | --- | --- |
| |  |  |  |  |  |  |  |  | | --- | --- | --- | --- | --- | --- | --- | --- | | **Overview** | **Package** | **Class** | **Use** | **Tree** | **Deprecated** | **Index** | **Help** | | |  |
| **PREV CLASS**   **NEXT CLASS** | **FRAMES**    **NO FRAMES**     **All Classes** |
| SUMMARY: NESTED | FIELD | CONSTR | METHOD | DETAIL: FIELD | CONSTR | METHOD |


---


## main Class DNACrypt

```
java.lang.Object
  main.DNACrypt
```

---

``` public class DNACrypt extends java.lang.Object ```

**Author:**
:   Dominik Heider

---

| **Constructor Summary** | |
| --- | --- |
| `DNACrypt()`             Creates an instance of DNACrypt |


| **Method Summary** | |
| --- | --- |
| `char[]` | `binaryCrypt(byte[] file, ForeignKey key, int correction)`             Encrypts the inputfile by using the binary encryption |
| `byte[]` | `binaryDecrypt(char[] genome, ForeignKey key, boolean inGenome, int correction)`             Decrypts a genome by using the binary decryption |
| `char[]` | `clellandCrypt(char[] text)`             Encrypts a text by using the Clelland method |
| `char[]` | `clellandDecrypt(char[] genome, boolean inGenome)`             Decrypts a genome by using the Clelland method |
| `java.util.Properties` | `defaultProperties()`             Sets Default Properties |
| `void` | `deleteKey(java.lang.String name, java.lang.String type, java.lang.String date)`             Deletes a key |
| `boolean` | `existUser(java.lang.String login)` |
| `void` | `exit()`             Exiting DNACrypt |
| `void` | `exportKey(java.lang.String name, java.lang.String type, java.lang.String date, java.io.File file)`             Exports a key to the file system |
| `User` | `findUser(java.lang.String login)`             Finds a User by login |
| `boolean` | `getbinaryFlag()` |
| `char[]` | `getGenome()` |
| `byte[]` | `getInputfile()` |
| `ForeignKey` | `getKey(java.lang.String name, java.lang.String type, java.lang.String date)` |
| `java.util.ArrayList<ForeignKey>` | `getKeyListe()` |
| `java.util.Properties` | `getProperties()` |
| `User` | `getUser()` |
| `void` | `importKey(java.io.File file)`             Imports a key from the file system |
| `void` | `loadFile(java.io.File file)`             Loads a file |
| `void` | `loadGenome(java.io.File file)`             Loads a genome |
| `OneTimePad` | `loadOneTimePad(java.io.File file)`             Loads a One Time Pad |
| `java.util.Properties` | `loadProperties()`             Loads the Property file |
| `UserManager` | `loadUserVerwaltung()`             Loads the UserManager |
| `void` | `loginUser(User user)`             Sets the current User to the given one |
| `void` | `logoutUser()`             Logs out the current User |
| `void` | `newKey(int keytype)`             Creates a new key |
| `void` | `newUser(java.lang.String name, java.lang.String surname, java.lang.String login, java.lang.String passwort)`             Creates a new User |
| `char[]` | `otpCrypt(int correction)`             Encrypts the inputfile by using a One Time Pad |
| `byte[]` | `otpDecrypt(OneTimePad otp, char[] genome, boolean inGenome, int correction)`             Decrypts a genome by using a One Time Pad |
| `char[]` | `reverse(char[] genome)`             Reverse transcribes a RNA sequence to DNA sequence |
| `void` | `saveFile(java.lang.String file, java.lang.Object obj)`             Saves a file |
| `void` | `saveOutput(java.io.File file)`             Saves the output to file system |
| `void` | `saveOutput(java.io.File file, java.lang.String output)`             Saves the output to file system |
| `void` | `setBinaryFlag(boolean setting)` |
| `void` | `setGenome(char[] genome)` |
| `void` | `setInputfile(byte[] inputfile)` |
| `void` | `setOutputfile(byte[] outputfile)` |
| `void` | `setUser(java.lang.String name, java.lang.String surname, java.lang.String passwort)`             Sets the attributes of the current User |
| `char[]` | `transcribe(char[] genome)`             Transcribes a DNA sequence |
| `char[]` | `translate(char[] genome)`             Translated a RNA sequence to a protein sequence |
| `void` | `updateProperties(java.lang.String text00, java.lang.String text01, java.lang.String text10, java.lang.String text11, java.lang.String textFILE, java.lang.String textGENOME, java.lang.String WDH_CONSTANT)`             Updates the properties |

| **Methods inherited from class java.lang.Object** |
| --- |
| `equals, getClass, hashCode, notify, notifyAll, toString, wait, wait, wait` |

| **Constructor Detail** |
| --- |

### DNACrypt

```
public DNACrypt()
```

:   Creates an instance of DNACrypt


| **Method Detail** |
| --- |

### defaultProperties

```
public java.util.Properties defaultProperties()
```

:   Sets Default Properties

    :   **Returns:**: the default Properties

---


### loadProperties

```
public java.util.Properties loadProperties()
```

:   Loads the Property file

    :   **Returns:**: the Property file

---


### saveFile

```
public void saveFile(java.lang.String file,
                     java.lang.Object obj)
```

:   Saves a file

    :   **Parameters:**: `file` - the target file: `obj` - the Object to save

---


### newUser

```
public void newUser(java.lang.String name,
                    java.lang.String surname,
                    java.lang.String login,
                    java.lang.String passwort)
```

:   Creates a new User

    :   **Parameters:**: `name` - the name of the user: `surname` - the surname of the user: `login` - the login of the user: `passwort` - the password of the user

---


### loginUser

```
public void loginUser(User user)
```

:   Sets the current User to the given one

    :   **Parameters:**: `user` - the current User

---


### existUser

```
public boolean existUser(java.lang.String login)
```

:   **Parameters:**: `login` - the login of the User **Returns:**: true, if User exists, otherwise false

---


### findUser

```
public User findUser(java.lang.String login)
```

:   Finds a User by login

    :   **Parameters:**: `login` - the login of the User **Returns:**: the User or null

---


### setUser

```
public void setUser(java.lang.String name,
                    java.lang.String surname,
                    java.lang.String passwort)
```

:   Sets the attributes of the current User

    :   **Parameters:**: `name` - the new name of the User: `surname` - the new surname of the User: `passwort` - the new Password of the User

---


### loadUserVerwaltung

```
public UserManager loadUserVerwaltung()
```

:   Loads the UserManager

    :   **Returns:**: the loaded UserManager

---


### loadOneTimePad

```
public OneTimePad loadOneTimePad(java.io.File file)
```

:   Loads a One Time Pad

    :   **Parameters:**: `file` - the source file **Returns:**: the loaded One Time Pad

---


### exit

```
public void exit()
```

:   Exiting DNACrypt

---


### saveOutput

```
public void saveOutput(java.io.File file,
                       java.lang.String output)
```

:   Saves the output to file system

    :   **Parameters:**: `file` - the target file: `the` - output to save

---


### saveOutput

```
public void saveOutput(java.io.File file)
```

:   Saves the output to file system

    :   **Parameters:**: `file` - the target file

---


### loadFile

```
public void loadFile(java.io.File file)
```

:   Loads a file

    :   **Parameters:**: `file` - the source file

---


### loadGenome

```
public void loadGenome(java.io.File file)
```

:   Loads a genome

    :   **Parameters:**: `file` - the source file

---


### getInputfile

```
public byte[] getInputfile()
```

:   **Returns:**: the inputfile (die eingelesene Datei)

---


### setInputfile

```
public void setInputfile(byte[] inputfile)
```

:   **Parameters:**: `inputfile` - The inputfile to set.

---


### getUser

```
public User getUser()
```

:   **Returns:**: Returns the user.

---


### logoutUser

```
public void logoutUser()
```

:   Logs out the current User

---


### getGenome

```
public char[] getGenome()
```

:   **Returns:**: Returns the genome.

---


### setGenome

```
public void setGenome(char[] genome)
```

:   **Parameters:**: `genome` - The genome to set.

---


### transcribe

```
public char[] transcribe(char[] genome)
```

:   Transcribes a DNA sequence

    :   **Parameters:**: `genome` - the DNA sequence **Returns:**: the RNA sequence

---


### translate

```
public char[] translate(char[] genome)
```

:   Translated a RNA sequence to a protein sequence

    :   **Parameters:**: `genome` - the RNA sequence **Returns:**: the protein sequence

---


### reverse

```
public char[] reverse(char[] genome)
```

:   Reverse transcribes a RNA sequence to DNA sequence

    :   **Parameters:**: `genome` - the RNA sequence **Returns:**: the DNA sequence

---


### clellandCrypt

```
public char[] clellandCrypt(char[] text)
                     throws java.lang.Exception
```

:   Encrypts a text by using the Clelland method

    :   **Parameters:**: `text` - the text to encode **Returns:**: the encoded text **Throws:**: `java.lang.Exception`

---


### otpCrypt

```
public char[] otpCrypt(int correction)
                throws java.lang.Exception
```

:   Encrypts the inputfile by using a One Time Pad

    :   **Returns:**: the encrypted file **Throws:**: `java.lang.Exception`

---


### binaryCrypt

```
public char[] binaryCrypt(byte[] file,
                          ForeignKey key,
                          int correction)
                   throws java.lang.Exception
```

:   Encrypts the inputfile by using the binary encryption

    :   **Parameters:**: `file` - the file to encode: `key` - the key for encryption or null **Returns:**: the encrypted file **Throws:**: `java.lang.Exception`

---


### clellandDecrypt

```
public char[] clellandDecrypt(char[] genome,
                              boolean inGenome)
                       throws java.lang.Exception
```

:   Decrypts a genome by using the Clelland method

    :   **Parameters:**: `genome` - the genome to decode: `inGenome` - true, if the text was encoded in a genome, otherwise false **Returns:**: the decoded text **Throws:**: `java.lang.Exception`

---


### otpDecrypt

```
public byte[] otpDecrypt(OneTimePad otp,
                         char[] genome,
                         boolean inGenome,
                         int correction)
                  throws java.lang.Exception
```

:   Decrypts a genome by using a One Time Pad

    :   **Parameters:**: `otp` - the One Time Pad: `genome` - the genome to decode: `inGenome` - true, if the text was encoded in a genome, otherwise false **Returns:**: the decrypted file **Throws:**: `java.lang.Exception`

---


### binaryDecrypt

```
public byte[] binaryDecrypt(char[] genome,
                            ForeignKey key,
                            boolean inGenome,
                            int correction)
                     throws java.lang.Exception
```

:   Decrypts a genome by using the binary decryption

    :   **Parameters:**: `genome` - the genome to decode: `key` - the key for decryption or null: `inGenome` - true, if the text was encoded in a genome, otherwise false **Returns:**: the decoded file **Throws:**: `java.lang.Exception`

---


### setOutputfile

```
public void setOutputfile(byte[] outputfile)
```

:   **Parameters:**: `outputfile` - The outputfile to set.

---


### setBinaryFlag

```
public void setBinaryFlag(boolean setting)
```

:   **Parameters:**: `setting` -

---


### getbinaryFlag

```
public boolean getbinaryFlag()
```

:   **Returns:**: the binary flag

---


### exportKey

```
public void exportKey(java.lang.String name,
                      java.lang.String type,
                      java.lang.String date,
                      java.io.File file)
```

:   Exports a key to the file system

    :   **Parameters:**: `name` - the name of the owner: `type` - the type of the key: `date` - the time of creation: `file` - the target file

---


### importKey

```
public void importKey(java.io.File file)
               throws java.lang.Exception
```

:   Imports a key from the file system

    :   **Parameters:**: `file` - the source file **Throws:**: `java.lang.Exception`

---


### getKey

```
public ForeignKey getKey(java.lang.String name,
                         java.lang.String type,
                         java.lang.String date)
```

:   **Parameters:**: `name` - the name of the owner: `type` - the type of the key: `date` - the time of creation **Returns:**: the key

---


### newKey

```
public void newKey(int keytype)
```

:   Creates a new key

    :   **Parameters:**: `keytype` - the type of the new key

---


### getKeyListe

```
public java.util.ArrayList<ForeignKey> getKeyListe()
```

:   **Returns:**: the keylist

---


### deleteKey

```
public void deleteKey(java.lang.String name,
                      java.lang.String type,
                      java.lang.String date)
```

:   Deletes a key

    :   **Parameters:**: `name` - the name of the owner: `type` - the type of the key: `date` - the time of creation

---


### getProperties

```
public java.util.Properties getProperties()
```

:   **Returns:**: the properties.

---


### updateProperties

```
public void updateProperties(java.lang.String text00,
                             java.lang.String text01,
                             java.lang.String text10,
                             java.lang.String text11,
                             java.lang.String textFILE,
                             java.lang.String textGENOME,
                             java.lang.String WDH_CONSTANT)
```

:   Updates the properties

    :   **Parameters:**: `text00` - the binary encryption for "00": `text01` - the binary encryption for "01": `text10` - the binary encryption for "10": `text11` - the binary encryption for "11"


---


|  |  |  |  |  |  |  |  |  |  |  |
| --- | --- | --- | --- | --- | --- | --- | --- | --- | --- | --- |
| |  |  |  |  |  |  |  |  | | --- | --- | --- | --- | --- | --- | --- | --- | | **Overview** | **Package** | **Class** | **Use** | **Tree** | **Deprecated** | **Index** | **Help** | | |  |
| **PREV CLASS**   **NEXT CLASS** | **FRAMES**    **NO FRAMES**     **All Classes** |
| SUMMARY: NESTED | FIELD | CONSTR | METHOD | DETAIL: FIELD | CONSTR | METHOD |


---
