# Supplementary material for: DNA-based watermarks using the DNA-Crypt algorithm
Source: BMC Bioinformatics. 2007 May 29;8:176. doi: 10.1186/1471-2105-8-176 (PMC1904243; doi:10.1186/1471-2105-8-176)
Supplement: Additional file 1 — The DNA-Crypt v.2. [file 1471-2105-8-176-S1.zip › help/doc/main/KeyManager.html]

KeyManager


|  |  |  |  |  |  |  |  |  |  |  |
| --- | --- | --- | --- | --- | --- | --- | --- | --- | --- | --- |
| |  |  |  |  |  |  |  |  | | --- | --- | --- | --- | --- | --- | --- | --- | | **Overview** | **Package** | **Class** | **Use** | **Tree** | **Deprecated** | **Index** | **Help** | | |  |
| **PREV CLASS**   **NEXT CLASS** | **FRAMES**    **NO FRAMES**     **All Classes** |
| SUMMARY: NESTED | FIELD | CONSTR | METHOD | DETAIL: FIELD | CONSTR | METHOD |


---


## main Class KeyManager

```
java.lang.Object
  main.KeyManager
```

**All Implemented Interfaces:**: java.io.Serializable

---

``` public class KeyManager extends java.lang.Object implements java.io.Serializable ```

**Author:**
:   Dominik

**See Also:**: Serialized Form

---

| **Constructor Summary** | |
| --- | --- |
| `KeyManager()`             Creates an instance of KeyManager |


| **Method Summary** | |
| --- | --- |
| `void` | `deleteKey(ForeignKey foreignKey)`             Deletes a key |
| `void` | `deleteKey(java.lang.String name, java.lang.String type, java.lang.String date)`             Deletes a key |
| `ForeignKey` | `find(java.lang.String name, java.lang.String type, java.lang.String date)`             Finds a key in the keylist |
| `java.util.ArrayList<ForeignKey>` | `getKeyListe()` |
| `void` | `newKey(ForeignKey foreignKey)`             Adds a key to the keylist |

| **Methods inherited from class java.lang.Object** |
| --- |
| `equals, getClass, hashCode, notify, notifyAll, toString, wait, wait, wait` |

| **Constructor Detail** |
| --- |

### KeyManager

```
public KeyManager()
```

:   Creates an instance of KeyManager


| **Method Detail** |
| --- |

### newKey

```
public void newKey(ForeignKey foreignKey)
            throws java.lang.Exception
```

:   Adds a key to the keylist

    :   **Parameters:**: `foreignKey` - the new key **Throws:**: `java.lang.Exception`

---


### find

```
public ForeignKey find(java.lang.String name,
                       java.lang.String type,
                       java.lang.String date)
```

:   Finds a key in the keylist

    :   **Parameters:**: `name` - the owner of the key: `type` - the type of the key: `date` - the time of creation **Returns:**: the key, otherwise null

---


### deleteKey

```
public void deleteKey(java.lang.String name,
                      java.lang.String type,
                      java.lang.String date)
```

:   Deletes a key

    :   **Parameters:**: `name` - the name of the owner: `type` - the type of the key: `date` - the time of creation

---


### deleteKey

```
public void deleteKey(ForeignKey foreignKey)
```

:   Deletes a key

    :   **Parameters:**: `foreignKey` - the key to delete

---


### getKeyListe

```
public java.util.ArrayList<ForeignKey> getKeyListe()
```

:   **Returns:**: Returns the keyListe.


---


|  |  |  |  |  |  |  |  |  |  |  |
| --- | --- | --- | --- | --- | --- | --- | --- | --- | --- | --- |
| |  |  |  |  |  |  |  |  | | --- | --- | --- | --- | --- | --- | --- | --- | | **Overview** | **Package** | **Class** | **Use** | **Tree** | **Deprecated** | **Index** | **Help** | | |  |
| **PREV CLASS**   **NEXT CLASS** | **FRAMES**    **NO FRAMES**     **All Classes** |
| SUMMARY: NESTED | FIELD | CONSTR | METHOD | DETAIL: FIELD | CONSTR | METHOD |


---
