# Supplementary material for: DNA-based watermarks using the DNA-Crypt algorithm
Source: BMC Bioinformatics. 2007 May 29;8:176. doi: 10.1186/1471-2105-8-176 (PMC1904243; doi:10.1186/1471-2105-8-176)
Supplement: Additional file 1 — The DNA-Crypt v.2. [file 1471-2105-8-176-S1.zip › help/doc/main/User.html]

User


|  |  |  |  |  |  |  |  |  |  |  |
| --- | --- | --- | --- | --- | --- | --- | --- | --- | --- | --- |
| |  |  |  |  |  |  |  |  | | --- | --- | --- | --- | --- | --- | --- | --- | | **Overview** | **Package** | **Class** | **Use** | **Tree** | **Deprecated** | **Index** | **Help** | | |  |
| **PREV CLASS**   **NEXT CLASS** | **FRAMES**    **NO FRAMES**     **All Classes** |
| SUMMARY: NESTED | FIELD | CONSTR | METHOD | DETAIL: FIELD | CONSTR | METHOD |


---


## main Class User

```
java.lang.Object
  main.User
```

**All Implemented Interfaces:**: java.io.Serializable

---

``` public class User extends java.lang.Object implements java.io.Serializable ```

**Author:**
:   Dominik

**See Also:**: Serialized Form

---

| **Constructor Summary** | |
| --- | --- |
| `User(java.lang.String name, java.lang.String surname, java.lang.String login, java.lang.String passwort)`             Creates an instance ot User |


| **Method Summary** | |
| --- | --- |
| `void` | `deleteKey(java.lang.String name2, java.lang.String type, java.lang.String date)`             Deletes a Key |
| `ForeignKey` | `getKey(java.lang.String name2, java.lang.String type, java.lang.String date)` |
| `java.util.ArrayList<ForeignKey>` | `getKeyListe()` |
| `KeyManager` | `getKeymanager()` |
| `java.lang.String` | `getLogin()` |
| `java.lang.String` | `getName()` |
| `java.lang.String` | `getPasswort()` |
| `java.lang.String` | `getVorname()` |
| `void` | `newKey(ForeignKey foreignKey)`             Adds a key to the KeyManager |
| `void` | `newKey(int keytype)`             Creates a new key in the KeyManager |
| `void` | `setLogin(java.lang.String login)` |
| `void` | `setName(java.lang.String name)` |
| `void` | `setPasswort(java.lang.String passwort)` |
| `void` | `setVorname(java.lang.String surname)` |

| **Methods inherited from class java.lang.Object** |
| --- |
| `equals, getClass, hashCode, notify, notifyAll, toString, wait, wait, wait` |

| **Constructor Detail** |
| --- |

### User

```
public User(java.lang.String name,
            java.lang.String surname,
            java.lang.String login,
            java.lang.String passwort)
```

:   Creates an instance ot User

    **Parameters:**: `name` - the name of the User: `surname` - the surname of the User: `login` - the Login of the User: `passwort` - the Password of the User


| **Method Detail** |
| --- |

### getName

```
public java.lang.String getName()
```

:   **Returns:**: Returns the name.

---


### setName

```
public void setName(java.lang.String name)
```

:   **Parameters:**: `name` - The name to set.

---


### getPasswort

```
public java.lang.String getPasswort()
```

:   **Returns:**: Returns the passwort.

---


### setPasswort

```
public void setPasswort(java.lang.String passwort)
```

:   **Parameters:**: `passwort` - The passwort to set.

---


### getLogin

```
public java.lang.String getLogin()
```

:   **Returns:**: Returns the login.

---


### setLogin

```
public void setLogin(java.lang.String login)
```

:   **Parameters:**: `login` - The login to set.

---


### getVorname

```
public java.lang.String getVorname()
```

:   **Returns:**: Returns the surname.

---


### setVorname

```
public void setVorname(java.lang.String surname)
```

:   **Parameters:**: `surname` - The surname to set.

---


### getKeymanager

```
public KeyManager getKeymanager()
```

:   **Returns:**: Returns the keymanager.

---


### getKeyListe

```
public java.util.ArrayList<ForeignKey> getKeyListe()
```

:   **Returns:**: the keylist of the KeyManager

---


### deleteKey

```
public void deleteKey(java.lang.String name2,
                      java.lang.String type,
                      java.lang.String date)
```

:   Deletes a Key

    :   **Parameters:**: `name2` - the name of the owner: `type` - the type of the key: `date` - the time of creation

---


### newKey

```
public void newKey(ForeignKey foreignKey)
            throws java.lang.Exception
```

:   Adds a key to the KeyManager

    :   **Parameters:**: `foreignKey` - the new key **Throws:**: `java.lang.Exception`

---


### newKey

```
public void newKey(int keytype)
```

:   Creates a new key in the KeyManager

    :   **Parameters:**: `keytype` - the type of the key

---


### getKey

```
public ForeignKey getKey(java.lang.String name2,
                         java.lang.String type,
                         java.lang.String date)
```

:   **Parameters:**: `name2` - the name of the key: `type` - the type of the key: `date` - the time of creation **Returns:**: the founded key


---


|  |  |  |  |  |  |  |  |  |  |  |
| --- | --- | --- | --- | --- | --- | --- | --- | --- | --- | --- |
| |  |  |  |  |  |  |  |  | | --- | --- | --- | --- | --- | --- | --- | --- | | **Overview** | **Package** | **Class** | **Use** | **Tree** | **Deprecated** | **Index** | **Help** | | |  |
| **PREV CLASS**   **NEXT CLASS** | **FRAMES**    **NO FRAMES**     **All Classes** |
| SUMMARY: NESTED | FIELD | CONSTR | METHOD | DETAIL: FIELD | CONSTR | METHOD |


---
