# Supplementary material for: DNA-based watermarks using the DNA-Crypt algorithm
Source: BMC Bioinformatics. 2007 May 29;8:176. doi: 10.1186/1471-2105-8-176 (PMC1904243; doi:10.1186/1471-2105-8-176)
Supplement: Additional file 1 — The DNA-Crypt v.2. [file 1471-2105-8-176-S1.zip › help/doc/main/UserManager.html]

UserManager


|  |  |  |  |  |  |  |  |  |  |  |
| --- | --- | --- | --- | --- | --- | --- | --- | --- | --- | --- |
| |  |  |  |  |  |  |  |  | | --- | --- | --- | --- | --- | --- | --- | --- | | **Overview** | **Package** | **Class** | **Use** | **Tree** | **Deprecated** | **Index** | **Help** | | |  |
| **PREV CLASS**   NEXT CLASS | **FRAMES**    **NO FRAMES**     **All Classes** |
| SUMMARY: NESTED | FIELD | CONSTR | METHOD | DETAIL: FIELD | CONSTR | METHOD |


---


## main Class UserManager

```
java.lang.Object
  main.UserManager
```

**All Implemented Interfaces:**: java.io.Serializable

---

``` public class UserManager extends java.lang.Object implements java.io.Serializable ```

The Class represents a Manager for the user.

**Author:**
:   Dominik Heider

**See Also:**: Serialized Form

---

| **Constructor Summary** | |
| --- | --- |
| `UserManager()`             Creates an instance of UserManager |


| **Method Summary** | |
| --- | --- |
| `void` | `deleteUser(java.lang.String login)`             Deletes a User |
| `boolean` | `existUser(java.lang.String login)` |
| `User` | `find(java.lang.String login)`             Finds a User by login |
| `void` | `newUser(User user)`             Adds a new User to the userlist |

| **Methods inherited from class java.lang.Object** |
| --- |
| `equals, getClass, hashCode, notify, notifyAll, toString, wait, wait, wait` |

| **Constructor Detail** |
| --- |

### UserManager

```
public UserManager()
```

:   Creates an instance of UserManager


| **Method Detail** |
| --- |

### newUser

```
public void newUser(User user)
```

:   Adds a new User to the userlist

    :   **Parameters:**: `user` - the new User

---


### find

```
public User find(java.lang.String login)
```

:   Finds a User by login

    :   **Parameters:**: `login` - the login of the User **Returns:**: the User with the login

---


### deleteUser

```
public void deleteUser(java.lang.String login)
```

:   Deletes a User

    :   **Parameters:**: `login` - the login of the User

---


### existUser

```
public boolean existUser(java.lang.String login)
```

:   **Parameters:**: `login` - the login of the User **Returns:**: false, if the user does not exist, otherwise true


---


|  |  |  |  |  |  |  |  |  |  |  |
| --- | --- | --- | --- | --- | --- | --- | --- | --- | --- | --- |
| |  |  |  |  |  |  |  |  | | --- | --- | --- | --- | --- | --- | --- | --- | | **Overview** | **Package** | **Class** | **Use** | **Tree** | **Deprecated** | **Index** | **Help** | | |  |
| **PREV CLASS**   NEXT CLASS | **FRAMES**    **NO FRAMES**     **All Classes** |
| SUMMARY: NESTED | FIELD | CONSTR | METHOD | DETAIL: FIELD | CONSTR | METHOD |


---
