# Supplementary material for: DNA-based watermarks using the DNA-Crypt algorithm
Source: BMC Bioinformatics. 2007 May 29;8:176. doi: 10.1186/1471-2105-8-176 (PMC1904243; doi:10.1186/1471-2105-8-176)
Supplement: Additional file 1 — The DNA-Crypt v.2. [file 1471-2105-8-176-S1.zip › help/doc/overview-tree.html]

Class Hierarchy


|  |  |  |  |  |  |  |  |  |  |  |
| --- | --- | --- | --- | --- | --- | --- | --- | --- | --- | --- |
| |  |  |  |  |  |  |  |  | | --- | --- | --- | --- | --- | --- | --- | --- | | **Overview** | Package | Class | Use | **Tree** | **Deprecated** | **Index** | **Help** | | |  |
| PREV   NEXT | **FRAMES**    **NO FRAMES**     **All Classes** |


---


## Hierarchy For All Packages

**Package Hierarchies:**: asymmetric, foreignKeys, genome, main, steg, symmetric

---

## Class Hierarchy

- java.lang.Object
  - symmetric.**AES**- steg.**AminoSteg**- genome.**Analyser**- steg.**BitCoding**- symmetric.**Blowfish**- main.**BrowserControl**- steg.**Clelland**- main.**DNACrypt**- foreignKeys.**ForeignAESBlowfishKey** (implements foreignKeys.ForeignKey, java.io.Serializable)- foreignKeys.**ForeignRSAKey** (implements foreignKeys.ForeignKey, java.io.Serializable)- genome.**GenomeOperator**- steg.**HammingCode** (implements steg.CorrectionCode)- main.**KeyManager** (implements java.io.Serializable)- steg.**NonCorrection** (implements steg.CorrectionCode)- symmetric.**OneTimePad** (implements java.io.Serializable)- asymmetric.**RSA**- main.**User** (implements java.io.Serializable)- main.**UserManager** (implements java.io.Serializable)- steg.**WDHC** (implements steg.CorrectionCode)

## Interface Hierarchy

- steg.**CorrectionCode**- java.io.Serializable
    - foreignKeys.**ForeignKey**

---


|  |  |  |  |  |  |  |  |  |  |  |
| --- | --- | --- | --- | --- | --- | --- | --- | --- | --- | --- |
| |  |  |  |  |  |  |  |  | | --- | --- | --- | --- | --- | --- | --- | --- | | **Overview** | Package | Class | Use | **Tree** | **Deprecated** | **Index** | **Help** | | |  |
| PREV   NEXT | **FRAMES**    **NO FRAMES**     **All Classes** |


---
