# Supplementary material for: DNA-based watermarks using the DNA-Crypt algorithm
Source: BMC Bioinformatics. 2007 May 29;8:176. doi: 10.1186/1471-2105-8-176 (PMC1904243; doi:10.1186/1471-2105-8-176)
Supplement: Additional file 1 — The DNA-Crypt v.2. [file 1471-2105-8-176-S1.zip › help/doc/serialized-form.html]

Serialized Form


|  |  |  |  |  |  |  |  |  |  |  |
| --- | --- | --- | --- | --- | --- | --- | --- | --- | --- | --- |
| |  |  |  |  |  |  |  |  | | --- | --- | --- | --- | --- | --- | --- | --- | | **Overview** | Package | Class | Use | **Tree** | **Deprecated** | **Index** | **Help** | | |  |
| PREV   NEXT | **FRAMES**    **NO FRAMES**     **All Classes** |


---


# Serialized Form


---

| **Package** **foreignKeys** |
| --- |

| **Class foreignKeys.ForeignAESBlowfishKey extends java.lang.Object implements Serializable** | |
| --- | --- |

**serialVersionUID:**-3352650794418866854L

| **Serialized Fields** |
| --- |

### name

```
java.lang.String name
```

:   The name of the owner of the key.

---

### type

```
java.lang.String type
```

:   the type of the key, AES or Blowfish

---

### aes

```
java.lang.String aes
```

---

### blowfish

```
java.lang.String blowfish
```

---

### date

```
java.util.Date date
```

:   the time of creation

---

### skeySpec

```
javax.crypto.spec.SecretKeySpec skeySpec
```

:   the Secret key

| **Class foreignKeys.ForeignRSAKey extends java.lang.Object implements Serializable** | |
| --- | --- |

**serialVersionUID:**-6653775059944003538L

| **Serialized Fields** |
| --- |

### name

```
java.lang.String name
```

:   The name of the owner of the key.

---

### type

```
java.lang.String type
```

:   the type of the key, RSA-Public or RSA-Private key

---

### date

```
java.util.Date date
```

:   the time of creation

---

### key

```
java.security.Key key
```

:   the Secret key

---

| **Package** **main** |
| --- |

| **Class main.KeyManager extends java.lang.Object implements Serializable** | |
| --- | --- |

**serialVersionUID:**6055025131206655427L

| **Serialized Fields** |
| --- |

### keyListe

```
java.util.ArrayList<E> keyListe
```

:   The list of all keys

| **Class main.User extends java.lang.Object implements Serializable** | |
| --- | --- |

**serialVersionUID:**-934927080830279651L

| **Serialized Fields** |
| --- |

### name

```
java.lang.String name
```

:   The name of the User

---

### surname

```
java.lang.String surname
```

:   The surname of the User

---

### login

```
java.lang.String login
```

:   The Login of the User

---

### passwort

```
java.lang.String passwort
```

:   The Password of the User

---

### keymanager

```
KeyManager keymanager
```

:   The KeyManager contains the keys of the User

---

### AESKey

```
int AESKey
```

---

### RSAKey

```
int RSAKey
```

---

### BlowfishKey

```
int BlowfishKey
```

| **Class main.UserManager extends java.lang.Object implements Serializable** | |
| --- | --- |

**serialVersionUID:**-2786180084330845933L

| **Serialized Fields** |
| --- |

### userListe

```
java.util.ArrayList<E> userListe
```

:   The list of all user

---

| **Package** **symmetric** |
| --- |

| **Class symmetric.OneTimePad extends java.lang.Object implements Serializable** | |
| --- | --- |

**serialVersionUID:**-1059061829394363287L

| **Serialized Fields** |
| --- |

### key

```
byte[] key
```

:   The byte array used for encryption or decryption

---


|  |  |  |  |  |  |  |  |  |  |  |
| --- | --- | --- | --- | --- | --- | --- | --- | --- | --- | --- |
| |  |  |  |  |  |  |  |  | | --- | --- | --- | --- | --- | --- | --- | --- | | **Overview** | Package | Class | Use | **Tree** | **Deprecated** | **Index** | **Help** | | |  |
| PREV   NEXT | **FRAMES**    **NO FRAMES**     **All Classes** |


---
