# Supplementary material for: DNA-based watermarks using the DNA-Crypt algorithm
Source: BMC Bioinformatics. 2007 May 29;8:176. doi: 10.1186/1471-2105-8-176 (PMC1904243; doi:10.1186/1471-2105-8-176)
Supplement: Additional file 1 — The DNA-Crypt v.2. [file 1471-2105-8-176-S1.zip › help/doc/steg/AminoSteg.html]

AminoSteg


|  |  |  |  |  |  |  |  |  |  |  |
| --- | --- | --- | --- | --- | --- | --- | --- | --- | --- | --- |
| |  |  |  |  |  |  |  |  | | --- | --- | --- | --- | --- | --- | --- | --- | | **Overview** | **Package** | **Class** | **Use** | **Tree** | **Deprecated** | **Index** | **Help** | | |  |
| PREV CLASS   **NEXT CLASS** | **FRAMES**    **NO FRAMES**     **All Classes** |
| SUMMARY: NESTED | FIELD | CONSTR | METHOD | DETAIL: FIELD | CONSTR | METHOD |


---


## steg Class AminoSteg

```
java.lang.Object
  steg.AminoSteg
```

---

``` public class AminoSteg extends java.lang.Object ```

**Author:**
:   Dominik

---

| **Field Summary** | |
| --- | --- |
| `int` | `MAXIMUM_FILE_SIZE`             The maximum sequence size which can be encrypted. |


| **Constructor Summary** | |
| --- | --- |
| `AminoSteg(DNACrypt dnacrypt)`             Creates an instance of AminoSteg |


| **Method Summary** | |
| --- | --- |
| `char[]` | `destretch(char[] genome)`             filters a sequence out of a given genome by reading the header. |
| `char[]` | `stretch(char[] genome, char[] sequence)`             hides a RNA sequence in a genome (RNA). |

| **Methods inherited from class java.lang.Object** |
| --- |
| `equals, getClass, hashCode, notify, notifyAll, toString, wait, wait, wait` |

| **Field Detail** |
| --- |

### MAXIMUM\_FILE\_SIZE

```
public int MAXIMUM_FILE_SIZE
```

:   The maximum sequence size which can be encrypted.
    Default: 2048 bytes


| **Constructor Detail** |
| --- |

### AminoSteg

```
public AminoSteg(DNACrypt dnacrypt)
```

:   Creates an instance of AminoSteg

    **Parameters:**: `dnacrypt` -


| **Method Detail** |
| --- |

### stretch

```
public char[] stretch(char[] genome,
                      char[] sequence)
               throws java.lang.Exception
```

:   hides a RNA sequence in a genome (RNA).
    It adds a header with contains the length of
    the RNA sequence. The maximum length is
    the MAXIMUM\_SEQUENCE\_SIZE.

    :   **Parameters:**: `genome` - the RNA sequence (genome): `sequence` - the RNA sequence to hide in the genome **Returns:**: the genome containing the RNA sequence **Throws:**: `java.lang.Exception`

---


### destretch

```
public char[] destretch(char[] genome)
                 throws java.lang.Exception
```

:   filters a sequence out of a given genome by reading the header.

    :   **Parameters:**: `genome` - the RNA sequence to filter **Returns:**: the filtered RNA sequence **Throws:**: `java.lang.Exception`


---


|  |  |  |  |  |  |  |  |  |  |  |
| --- | --- | --- | --- | --- | --- | --- | --- | --- | --- | --- |
| |  |  |  |  |  |  |  |  | | --- | --- | --- | --- | --- | --- | --- | --- | | **Overview** | **Package** | **Class** | **Use** | **Tree** | **Deprecated** | **Index** | **Help** | | |  |
| PREV CLASS   **NEXT CLASS** | **FRAMES**    **NO FRAMES**     **All Classes** |
| SUMMARY: NESTED | FIELD | CONSTR | METHOD | DETAIL: FIELD | CONSTR | METHOD |


---
