# Supplementary material for: DNA-based watermarks using the DNA-Crypt algorithm
Source: BMC Bioinformatics. 2007 May 29;8:176. doi: 10.1186/1471-2105-8-176 (PMC1904243; doi:10.1186/1471-2105-8-176)
Supplement: Additional file 1 — The DNA-Crypt v.2. [file 1471-2105-8-176-S1.zip › help/doc/steg/BitCoding.html]

BitCoding


|  |  |  |  |  |  |  |  |  |  |  |
| --- | --- | --- | --- | --- | --- | --- | --- | --- | --- | --- |
| |  |  |  |  |  |  |  |  | | --- | --- | --- | --- | --- | --- | --- | --- | | **Overview** | **Package** | **Class** | **Use** | **Tree** | **Deprecated** | **Index** | **Help** | | |  |
| **PREV CLASS**   **NEXT CLASS** | **FRAMES**    **NO FRAMES**     **All Classes** |
| SUMMARY: NESTED | FIELD | CONSTR | METHOD | DETAIL: FIELD | CONSTR | METHOD |


---


## steg Class BitCoding

```
java.lang.Object
  steg.BitCoding
```

---

``` public class BitCoding extends java.lang.Object ```

**Author:**
:   Dominik

---

| **Constructor Summary** | |
| --- | --- |
| `BitCoding(DNACrypt dnacrypt)`             Creates an instance of BitCoding |


| **Method Summary** | |
| --- | --- |
| `byte[]` | `decode(char[] genome)`             Filters a file out of a RNA sequence |
| `char[]` | `encode(byte[] file)`             Encodes a given byte array into an RNA sequence |

| **Methods inherited from class java.lang.Object** |
| --- |
| `equals, getClass, hashCode, notify, notifyAll, toString, wait, wait, wait` |

| **Constructor Detail** |
| --- |

### BitCoding

```
public BitCoding(DNACrypt dnacrypt)
```

:   Creates an instance of BitCoding

    **Parameters:**: `dnacrypt` -


| **Method Detail** |
| --- |

### encode

```
public char[] encode(byte[] file)
```

:   Encodes a given byte array into an RNA sequence

    :   **Parameters:**: `file` - the byte array to encode **Returns:**: the RNA sequence which contains the encoded file

---


### decode

```
public byte[] decode(char[] genome)
```

:   Filters a file out of a RNA sequence

    :   **Parameters:**: `genome` - the RNA sequence **Returns:**: the filtered byte array


---


|  |  |  |  |  |  |  |  |  |  |  |
| --- | --- | --- | --- | --- | --- | --- | --- | --- | --- | --- |
| |  |  |  |  |  |  |  |  | | --- | --- | --- | --- | --- | --- | --- | --- | | **Overview** | **Package** | **Class** | **Use** | **Tree** | **Deprecated** | **Index** | **Help** | | |  |
| **PREV CLASS**   **NEXT CLASS** | **FRAMES**    **NO FRAMES**     **All Classes** |
| SUMMARY: NESTED | FIELD | CONSTR | METHOD | DETAIL: FIELD | CONSTR | METHOD |


---
