# Supplementary material for: DNA-based watermarks using the DNA-Crypt algorithm
Source: BMC Bioinformatics. 2007 May 29;8:176. doi: 10.1186/1471-2105-8-176 (PMC1904243; doi:10.1186/1471-2105-8-176)
Supplement: Additional file 1 — The DNA-Crypt v.2. [file 1471-2105-8-176-S1.zip › help/doc/steg/class-use/AminoSteg.html]

Uses of Class steg.AminoSteg


|  |  |  |  |  |  |  |  |  |  |  |
| --- | --- | --- | --- | --- | --- | --- | --- | --- | --- | --- |
| |  |  |  |  |  |  |  |  | | --- | --- | --- | --- | --- | --- | --- | --- | | **Overview** | **Package** | **Class** | **Use** | **Tree** | **Deprecated** | **Index** | **Help** | | |  |
| PREV   NEXT | **FRAMES**    **NO FRAMES**     **All Classes** |


---


## **Uses of Class steg.AminoSteg**

No usage of steg.AminoSteg

---


|  |  |  |  |  |  |  |  |  |  |  |
| --- | --- | --- | --- | --- | --- | --- | --- | --- | --- | --- |
| |  |  |  |  |  |  |  |  | | --- | --- | --- | --- | --- | --- | --- | --- | | **Overview** | **Package** | **Class** | **Use** | **Tree** | **Deprecated** | **Index** | **Help** | | |  |
| PREV   NEXT | **FRAMES**    **NO FRAMES**     **All Classes** |


---
