# Supplementary material for: DNA-based watermarks using the DNA-Crypt algorithm
Source: BMC Bioinformatics. 2007 May 29;8:176. doi: 10.1186/1471-2105-8-176 (PMC1904243; doi:10.1186/1471-2105-8-176)
Supplement: Additional file 1 — The DNA-Crypt v.2. [file 1471-2105-8-176-S1.zip › help/doc/steg/class-use/CorrectionCode.html]

Uses of Interface steg.CorrectionCode


|  |  |  |  |  |  |  |  |  |  |  |
| --- | --- | --- | --- | --- | --- | --- | --- | --- | --- | --- |
| |  |  |  |  |  |  |  |  | | --- | --- | --- | --- | --- | --- | --- | --- | | **Overview** | **Package** | **Class** | **Use** | **Tree** | **Deprecated** | **Index** | **Help** | | |  |
| PREV   NEXT | **FRAMES**    **NO FRAMES**     **All Classes** |


---


## **Uses of Interface steg.CorrectionCode**

| Packages that use CorrectionCode | |
| --- | --- |
| **steg** |  |

| Uses of CorrectionCode in steg | |
| --- | --- |

| Classes in steg that implement CorrectionCode | |
| --- | --- |
| `class` | `HammingCode`             The Class encode/decodes a byte array with the 8/4 Hamming Code to correct one bit failure. |
| `class` | `NonCorrection`             The Class uses the identity function |
| `class` | `WDHC`             The Class encode/decodes a byte array with the WDH-Code |

---


|  |  |  |  |  |  |  |  |  |  |  |
| --- | --- | --- | --- | --- | --- | --- | --- | --- | --- | --- |
| |  |  |  |  |  |  |  |  | | --- | --- | --- | --- | --- | --- | --- | --- | | **Overview** | **Package** | **Class** | **Use** | **Tree** | **Deprecated** | **Index** | **Help** | | |  |
| PREV   NEXT | **FRAMES**    **NO FRAMES**     **All Classes** |


---
