# Supplementary material for: DNA-based watermarks using the DNA-Crypt algorithm
Source: BMC Bioinformatics. 2007 May 29;8:176. doi: 10.1186/1471-2105-8-176 (PMC1904243; doi:10.1186/1471-2105-8-176)
Supplement: Additional file 1 — The DNA-Crypt v.2. [file 1471-2105-8-176-S1.zip › help/doc/steg/Clelland.html]

Clelland


|  |  |  |  |  |  |  |  |  |  |  |
| --- | --- | --- | --- | --- | --- | --- | --- | --- | --- | --- |
| |  |  |  |  |  |  |  |  | | --- | --- | --- | --- | --- | --- | --- | --- | | **Overview** | **Package** | **Class** | **Use** | **Tree** | **Deprecated** | **Index** | **Help** | | |  |
| **PREV CLASS**   **NEXT CLASS** | **FRAMES**    **NO FRAMES**     **All Classes** |
| SUMMARY: NESTED | FIELD | CONSTR | METHOD | DETAIL: FIELD | CONSTR | METHOD |


---


## steg Class Clelland

```
java.lang.Object
  steg.Clelland
```

---

``` public class Clelland extends java.lang.Object ```

The Class encode/decodes a character array into a RNA sequence

**Author:**
:   Dominik Heider

---

| **Constructor Summary** | |
| --- | --- |
| `Clelland()` |


| **Method Summary** | |
| --- | --- |
| `char[]` | `decode(char[] genome)`             Decodes a character array out of a RNA sequence |
| `char[]` | `encode(char[] text)`             Encodes a character array into a RNA sequence |

| **Methods inherited from class java.lang.Object** |
| --- |
| `equals, getClass, hashCode, notify, notifyAll, toString, wait, wait, wait` |

| **Constructor Detail** |
| --- |

### Clelland

```
public Clelland()
```


| **Method Detail** |
| --- |

### encode

```
public char[] encode(char[] text)
```

:   Encodes a character array into a RNA sequence

    :   **Parameters:**: `text` - the character array to encode **Returns:**: the RNA sequence containing the encoded character array

---


### decode

```
public char[] decode(char[] genome)
```

:   Decodes a character array out of a RNA sequence

    :   **Parameters:**: `genome` - the RNA sequence **Returns:**: the decoded character array


---


|  |  |  |  |  |  |  |  |  |  |  |
| --- | --- | --- | --- | --- | --- | --- | --- | --- | --- | --- |
| |  |  |  |  |  |  |  |  | | --- | --- | --- | --- | --- | --- | --- | --- | | **Overview** | **Package** | **Class** | **Use** | **Tree** | **Deprecated** | **Index** | **Help** | | |  |
| **PREV CLASS**   **NEXT CLASS** | **FRAMES**    **NO FRAMES**     **All Classes** |
| SUMMARY: NESTED | FIELD | CONSTR | METHOD | DETAIL: FIELD | CONSTR | METHOD |


---
