# Supplementary material for: DNA-based watermarks using the DNA-Crypt algorithm
Source: BMC Bioinformatics. 2007 May 29;8:176. doi: 10.1186/1471-2105-8-176 (PMC1904243; doi:10.1186/1471-2105-8-176)
Supplement: Additional file 1 — The DNA-Crypt v.2. [file 1471-2105-8-176-S1.zip › help/doc/steg/CorrectionCode.html]

CorrectionCode


|  |  |  |  |  |  |  |  |  |  |  |
| --- | --- | --- | --- | --- | --- | --- | --- | --- | --- | --- |
| |  |  |  |  |  |  |  |  | | --- | --- | --- | --- | --- | --- | --- | --- | | **Overview** | **Package** | **Class** | **Use** | **Tree** | **Deprecated** | **Index** | **Help** | | |  |
| **PREV CLASS**   **NEXT CLASS** | **FRAMES**    **NO FRAMES**     **All Classes** |
| SUMMARY: NESTED | FIELD | CONSTR | METHOD | DETAIL: FIELD | CONSTR | METHOD |


---


## steg Interface CorrectionCode

**All Known Implementing Classes:**: HammingCode, NonCorrection, WDHC

---

``` public interface CorrectionCode ```

---

| **Method Summary** | |
| --- | --- |
| `byte[]` | `decode(byte[] seq)`             Decodes a bytearray |
| `byte[]` | `encode(byte[] seq)`             Encodes a bytearray |

| **Method Detail** |
| --- |

### encode

```
byte[] encode(byte[] seq)
```

:   Encodes a bytearray

    :   **Parameters:**: `seq` - the bytearray to encode **Returns:**: the encoded bytearray

---


### decode

```
byte[] decode(byte[] seq)
```

:   Decodes a bytearray

    :   **Parameters:**: `seq` - the bytearray to decode **Returns:**: the decoded bytearray


---


|  |  |  |  |  |  |  |  |  |  |  |
| --- | --- | --- | --- | --- | --- | --- | --- | --- | --- | --- |
| |  |  |  |  |  |  |  |  | | --- | --- | --- | --- | --- | --- | --- | --- | | **Overview** | **Package** | **Class** | **Use** | **Tree** | **Deprecated** | **Index** | **Help** | | |  |
| **PREV CLASS**   **NEXT CLASS** | **FRAMES**    **NO FRAMES**     **All Classes** |
| SUMMARY: NESTED | FIELD | CONSTR | METHOD | DETAIL: FIELD | CONSTR | METHOD |


---
