# Supplementary material for: DNA-based watermarks using the DNA-Crypt algorithm
Source: BMC Bioinformatics. 2007 May 29;8:176. doi: 10.1186/1471-2105-8-176 (PMC1904243; doi:10.1186/1471-2105-8-176)
Supplement: Additional file 1 — The DNA-Crypt v.2. [file 1471-2105-8-176-S1.zip › help/doc/steg/HammingCode.html]

HammingCode


|  |  |  |  |  |  |  |  |  |  |  |
| --- | --- | --- | --- | --- | --- | --- | --- | --- | --- | --- |
| |  |  |  |  |  |  |  |  | | --- | --- | --- | --- | --- | --- | --- | --- | | **Overview** | **Package** | **Class** | **Use** | **Tree** | **Deprecated** | **Index** | **Help** | | |  |
| **PREV CLASS**   **NEXT CLASS** | **FRAMES**    **NO FRAMES**     **All Classes** |
| SUMMARY: NESTED | FIELD | CONSTR | METHOD | DETAIL: FIELD | CONSTR | METHOD |


---


## steg Class HammingCode

```
java.lang.Object
  steg.HammingCode
```

**All Implemented Interfaces:**: CorrectionCode

---

``` public class HammingCode extends java.lang.Object implements CorrectionCode ```

The Class encode/decodes a byte array with the 8/4 Hamming Code
to correct one bit failure.

**Author:**
:   Dominik Heider

---

| **Constructor Summary** | |
| --- | --- |
| `HammingCode()` |


| **Method Summary** | |
| --- | --- |
| `byte` | `decode(byte seq)`             Decodes a byte h7, h6, h5, h4, h3, h2, h1, h0 p = h7 ^ h6 ^ h5 ^ h4 ^ h3 ^ h2 ^ h1 ^ h0 c0 = h7 ^ h5 ^ h1 ^ h0 c1 = h7 ^ h3 ^ h2 ^ h1 c2 = h5 ^ h4 ^ h3 ^ h1 If the parity, p, is correct (equal to 1) then either 0 or 2 errors occurred. |
| `byte[]` | `decode(byte[] seq)`             Decodes a byte array |
| `byte` | `encode(byte seq)`             The first four bits are used. |
| `byte[]` | `encode(byte[] seq)`             Encodes a byte array |

| **Methods inherited from class java.lang.Object** |
| --- |
| `equals, getClass, hashCode, notify, notifyAll, toString, wait, wait, wait` |

| **Constructor Detail** |
| --- |

### HammingCode

```
public HammingCode()
```


| **Method Detail** |
| --- |

### encode

```
public byte[] encode(byte[] seq)
```

:   Encodes a byte array

    :   **Specified by:**: `encode` in interface `CorrectionCode`
    :   **Parameters:**: `seq` - the byte array to encode **Returns:**: the encoded byte array

---


### decode

```
public byte[] decode(byte[] seq)
```

:   Decodes a byte array

    :   **Specified by:**: `decode` in interface `CorrectionCode`
    :   **Parameters:**: `seq` - the byte array to decode **Returns:**: the decoded byte array

---


### encode

```
public byte encode(byte seq)
```

:   The first four bits are used.
    b3, b2, b1, b0
    The Output is a byte as follows
    b3, b3^b2^b1, b2, !b2^b1^b0, b1, !b3^b1^b0, b0, !b3^b2^b0

    :   **Parameters:**: `seq` - the byte to encode **Returns:**: the encoded byte

---


### decode

```
public byte decode(byte seq)
            throws java.lang.Exception
```

:   Decodes a byte
    h7, h6, h5, h4, h3, h2, h1, h0
    p = h7 ^ h6 ^ h5 ^ h4 ^ h3 ^ h2 ^ h1 ^ h0
    c0 = h7 ^ h5 ^ h1 ^ h0
    c1 = h7 ^ h3 ^ h2 ^ h1
    c2 = h5 ^ h4 ^ h3 ^ h1
    If the parity, p, is correct (equal to 1) then either 0 or 2 errors occurred.
    If all the check bits, c0, c1, c2 are correct (equal to 1) then the byte was
    received intact, (no errors) otherwise it was damaged beyond repair (two errors).
    If p is 0, then there was a single bit error which can be recovered:
    c0 c1 c2 meaning
    1 1 1 error in bit h6
    1 1 0 error in bit h4
    1 0 1 error in bit h2
    0 1 1 error in bit h0
    0 0 1 error in bit h7
    0 1 0 error in bit h5
    1 0 0 error in bit h3
    0 0 0 error in bit h1
    The erroneous bit should be flipped. Note that there is actually no need to fix
    errors in bits h6, h4, h2 and h0 since they are not used in the decoded byte.
    After flipping bits if necessary, the decoded byte is then:
    h7, h5, h3, h1

    :   **Parameters:**: `seq` - the byte to decode **Returns:**: the decoded byte **Throws:**: `java.lang.Exception`


---


|  |  |  |  |  |  |  |  |  |  |  |
| --- | --- | --- | --- | --- | --- | --- | --- | --- | --- | --- |
| |  |  |  |  |  |  |  |  | | --- | --- | --- | --- | --- | --- | --- | --- | | **Overview** | **Package** | **Class** | **Use** | **Tree** | **Deprecated** | **Index** | **Help** | | |  |
| **PREV CLASS**   **NEXT CLASS** | **FRAMES**    **NO FRAMES**     **All Classes** |
| SUMMARY: NESTED | FIELD | CONSTR | METHOD | DETAIL: FIELD | CONSTR | METHOD |


---
