# Supplementary material for: DNA-based watermarks using the DNA-Crypt algorithm
Source: BMC Bioinformatics. 2007 May 29;8:176. doi: 10.1186/1471-2105-8-176 (PMC1904243; doi:10.1186/1471-2105-8-176)
Supplement: Additional file 1 — The DNA-Crypt v.2. [file 1471-2105-8-176-S1.zip › help/doc/steg/NonCorrection.html]

NonCorrection


|  |  |  |  |  |  |  |  |  |  |  |
| --- | --- | --- | --- | --- | --- | --- | --- | --- | --- | --- |
| |  |  |  |  |  |  |  |  | | --- | --- | --- | --- | --- | --- | --- | --- | | **Overview** | **Package** | **Class** | **Use** | **Tree** | **Deprecated** | **Index** | **Help** | | |  |
| **PREV CLASS**   **NEXT CLASS** | **FRAMES**    **NO FRAMES**     **All Classes** |
| SUMMARY: NESTED | FIELD | CONSTR | METHOD | DETAIL: FIELD | CONSTR | METHOD |


---


## steg Class NonCorrection

```
java.lang.Object
  steg.NonCorrection
```

**All Implemented Interfaces:**: CorrectionCode

---

``` public class NonCorrection extends java.lang.Object implements CorrectionCode ```

The Class uses the identity function

**Author:**
:   Dominik Heider

---

| **Constructor Summary** | |
| --- | --- |
| `NonCorrection()` |


| **Method Summary** | |
| --- | --- |
| `byte[]` | `decode(byte[] seq)`             Decodes a bytearray |
| `byte[]` | `encode(byte[] seq)`             Encodes a bytearray |

| **Methods inherited from class java.lang.Object** |
| --- |
| `equals, getClass, hashCode, notify, notifyAll, toString, wait, wait, wait` |

| **Constructor Detail** |
| --- |

### NonCorrection

```
public NonCorrection()
```


| **Method Detail** |
| --- |

### encode

```
public byte[] encode(byte[] seq)
```

:   **Description copied from interface: `CorrectionCode`**
:   Encodes a bytearray

    :   **Specified by:**: `encode` in interface `CorrectionCode`
    :   **Parameters:**: `seq` - the bytearray to encode **Returns:**: the encoded bytearray

---


### decode

```
public byte[] decode(byte[] seq)
```

:   **Description copied from interface: `CorrectionCode`**
:   Decodes a bytearray

    :   **Specified by:**: `decode` in interface `CorrectionCode`
    :   **Parameters:**: `seq` - the bytearray to decode **Returns:**: the decoded bytearray


---


|  |  |  |  |  |  |  |  |  |  |  |
| --- | --- | --- | --- | --- | --- | --- | --- | --- | --- | --- |
| |  |  |  |  |  |  |  |  | | --- | --- | --- | --- | --- | --- | --- | --- | | **Overview** | **Package** | **Class** | **Use** | **Tree** | **Deprecated** | **Index** | **Help** | | |  |
| **PREV CLASS**   **NEXT CLASS** | **FRAMES**    **NO FRAMES**     **All Classes** |
| SUMMARY: NESTED | FIELD | CONSTR | METHOD | DETAIL: FIELD | CONSTR | METHOD |


---
