# Supplementary material for: DNA-based watermarks using the DNA-Crypt algorithm
Source: BMC Bioinformatics. 2007 May 29;8:176. doi: 10.1186/1471-2105-8-176 (PMC1904243; doi:10.1186/1471-2105-8-176)
Supplement: Additional file 1 — The DNA-Crypt v.2. [file 1471-2105-8-176-S1.zip › help/doc/steg/WDHC.html]

WDHC


|  |  |  |  |  |  |  |  |  |  |  |
| --- | --- | --- | --- | --- | --- | --- | --- | --- | --- | --- |
| |  |  |  |  |  |  |  |  | | --- | --- | --- | --- | --- | --- | --- | --- | | **Overview** | **Package** | **Class** | **Use** | **Tree** | **Deprecated** | **Index** | **Help** | | |  |
| **PREV CLASS**   NEXT CLASS | **FRAMES**    **NO FRAMES**     **All Classes** |
| SUMMARY: NESTED | FIELD | CONSTR | METHOD | DETAIL: FIELD | CONSTR | METHOD |


---


## steg Class WDHC

```
java.lang.Object
  steg.WDHC
```

**All Implemented Interfaces:**: CorrectionCode

---

``` public class WDHC extends java.lang.Object implements CorrectionCode ```

The Class encode/decodes a byte array with the WDH-Code

**Author:**
:   Dominik Heider

---

| **Constructor Summary** | |
| --- | --- |
| `WDHC(int constant)` |


| **Method Summary** | |
| --- | --- |
| `byte[]` | `decode(byte[] seq)`             Decodes a byte array |
| `byte[]` | `encode(byte[] seq)`             Encodes a byte array |

| **Methods inherited from class java.lang.Object** |
| --- |
| `equals, getClass, hashCode, notify, notifyAll, toString, wait, wait, wait` |

| **Constructor Detail** |
| --- |

### WDHC

```
public WDHC(int constant)
```


| **Method Detail** |
| --- |

### encode

```
public byte[] encode(byte[] seq)
```

:   Encodes a byte array

    :   **Specified by:**: `encode` in interface `CorrectionCode`
    :   **Parameters:**: `seq` - the byte array to encode **Returns:**: the encoded byte array

---


### decode

```
public byte[] decode(byte[] seq)
```

:   Decodes a byte array

    :   **Specified by:**: `decode` in interface `CorrectionCode`
    :   **Parameters:**: `seq` - the byte array to decode **Returns:**: the decoded byte array


---


|  |  |  |  |  |  |  |  |  |  |  |
| --- | --- | --- | --- | --- | --- | --- | --- | --- | --- | --- |
| |  |  |  |  |  |  |  |  | | --- | --- | --- | --- | --- | --- | --- | --- | | **Overview** | **Package** | **Class** | **Use** | **Tree** | **Deprecated** | **Index** | **Help** | | |  |
| **PREV CLASS**   NEXT CLASS | **FRAMES**    **NO FRAMES**     **All Classes** |
| SUMMARY: NESTED | FIELD | CONSTR | METHOD | DETAIL: FIELD | CONSTR | METHOD |


---
