# Supplementary material for: DNA-based watermarks using the DNA-Crypt algorithm
Source: BMC Bioinformatics. 2007 May 29;8:176. doi: 10.1186/1471-2105-8-176 (PMC1904243; doi:10.1186/1471-2105-8-176)
Supplement: Additional file 1 — The DNA-Crypt v.2. [file 1471-2105-8-176-S1.zip › help/doc/symmetric/OneTimePad.html]

OneTimePad


|  |  |  |  |  |  |  |  |  |  |  |
| --- | --- | --- | --- | --- | --- | --- | --- | --- | --- | --- |
| |  |  |  |  |  |  |  |  | | --- | --- | --- | --- | --- | --- | --- | --- | | **Overview** | **Package** | **Class** | **Use** | **Tree** | **Deprecated** | **Index** | **Help** | | |  |
| **PREV CLASS**   NEXT CLASS | **FRAMES**    **NO FRAMES**     **All Classes** |
| SUMMARY: NESTED | FIELD | CONSTR | METHOD | DETAIL: FIELD | CONSTR | METHOD |


---


## symmetric Class OneTimePad

```
java.lang.Object
  symmetric.OneTimePad
```

**All Implemented Interfaces:**: java.io.Serializable

---

``` public class OneTimePad extends java.lang.Object implements java.io.Serializable ```

**Author:**
:   Dominik

**See Also:**: Serialized Form

---

| **Constructor Summary** | |
| --- | --- |
| `OneTimePad(int size)`             Creates a new One Time Pad |


| **Method Summary** | |
| --- | --- |
| `byte[]` | `crypt(byte[] seq)`             Encrypts or Decrypts a byte array by using XOR |
| `byte[]` | `getKey()` |

| **Methods inherited from class java.lang.Object** |
| --- |
| `equals, getClass, hashCode, notify, notifyAll, toString, wait, wait, wait` |

| **Constructor Detail** |
| --- |

### OneTimePad

```
public OneTimePad(int size)
```

:   Creates a new One Time Pad

    **Parameters:**: `size` - the size of the One Time Pad


| **Method Detail** |
| --- |

### crypt

```
public byte[] crypt(byte[] seq)
```

:   Encrypts or Decrypts a byte array by using XOR

    :   **Parameters:**: `seq` - the byte array to encode/decode **Returns:**: the encoded/decoded byte array

---


### getKey

```
public byte[] getKey()
```

:   **Returns:**: Returns the key.


---


|  |  |  |  |  |  |  |  |  |  |  |
| --- | --- | --- | --- | --- | --- | --- | --- | --- | --- | --- |
| |  |  |  |  |  |  |  |  | | --- | --- | --- | --- | --- | --- | --- | --- | | **Overview** | **Package** | **Class** | **Use** | **Tree** | **Deprecated** | **Index** | **Help** | | |  |
| **PREV CLASS**   NEXT CLASS | **FRAMES**    **NO FRAMES**     **All Classes** |
| SUMMARY: NESTED | FIELD | CONSTR | METHOD | DETAIL: FIELD | CONSTR | METHOD |


---
