# Supplementary material for: DNA-based watermarks using the DNA-Crypt algorithm
Source: BMC Bioinformatics. 2007 May 29;8:176. doi: 10.1186/1471-2105-8-176 (PMC1904243; doi:10.1186/1471-2105-8-176)
Supplement: Additional file 1 — The DNA-Crypt v.2. [file 1471-2105-8-176-S1.zip › help/help1.html]

DNA-Crypt  
  
1. Introduction

Information is the most wanted trade good of our time. In order to protect this   
information, you have to encrypt your information.  
DNA-Crypt is a program that offers the functionality to do so, but in a very special way -   
it encodes your files into DNA sequences. These sequences can be transfered to   
bacteria or other organisms without influencing them. You can use cryptographic keys,   
like RSA, AES or Blowfish, or, for maximum security, you can use a one time pad with   
DNA Crypt.

Previous - Next
