# Supplementary material for: DNA-based watermarks using the DNA-Crypt algorithm
Source: BMC Bioinformatics. 2007 May 29;8:176. doi: 10.1186/1471-2105-8-176 (PMC1904243; doi:10.1186/1471-2105-8-176)
Supplement: Additional file 1 — The DNA-Crypt v.2. [file 1471-2105-8-176-S1.zip › help/help2.html]

DNA-Crypt  
  
2. First steps

First you have to create a user account for DNA-Crypt.   
After doing so, you can log-in to the system.   
DNA-Crypt automaticly creates RSA, AES and a Blowfish key for every new user.  
The following screenshots show how you can easily load a file or type your own text   
in the file panel.
  
  
  
  
  
  
  
If you want, you can load a genome too.  
  
  
  
  
Now you can encrypt your file or text.  
  
  
  
  
The encoded DNA sequence can be found in the output panel   
  
  
  
  
If you want to decrypt a file from a DNA sequence, just copy the sequence in the file panel and decrypt.

  
  
Previous - Next
