# Supplementary material for: DNA-based watermarks using the DNA-Crypt algorithm
Source: BMC Bioinformatics. 2007 May 29;8:176. doi: 10.1186/1471-2105-8-176 (PMC1904243; doi:10.1186/1471-2105-8-176)
Supplement: Additional file 1 — The DNA-Crypt v.2. [file 1471-2105-8-176-S1.zip › help/help3.html]

DNA-Crypt  
  
3. The menus

3.1 The File-Menu  
3.2 The User-Menu  
3.3 The Genome-Menu  
3.1 The Key-Menu

  
  
Previous - Next
