# Supplementary material for: DNA-based watermarks using the DNA-Crypt algorithm
Source: BMC Bioinformatics. 2007 May 29;8:176. doi: 10.1186/1471-2105-8-176 (PMC1904243; doi:10.1186/1471-2105-8-176)
Supplement: Additional file 1 — The DNA-Crypt v.2. [file 1471-2105-8-176-S1.zip › help/help31.html]

DNA-Crypt  
  
3. The menus

**3.1 The File-Menu**  
  
**3.1.1 How to create an empty text**  
  
To type in a text for encryption or decryption use **Menu->File->New**   
or the mnemonic **CTRL+N**.  
  
  
  
  
**3.1.2 How to open a binary file**  
  
To open a binary file for encryption use **Menu->File->Open...**   
or the mnemonic **CTRL+O**.  
  
  
  
  
**3.1.3 How encrypt a text or a file**  
  
To encrypt a binary file or a text, use **Menu->File->Crypt**.  
DNA-Crypt recognize either a file or a text have to be encrypted and opens   
 the Clellandencryption Dialog or the Binaryencryption Dialog.  
In the Binaryencryption Dialog you have to choose if you want to use a encryption   
key, a One Time Pad or no further cryptographic encryption.  
  
  
  
  
  
  
  
  
**3.1.4 How decrypt a text or a file**  
  
To decrypt a binary file or a text from a sequence use **Menu->File->Decrypt**.  
Now you have to know, whether a text or a file was enrypted in the sequence.   
Further you have to know if it was encrypted in a genome or not.
  
In kind of binary encryption you have to choose the decryption key.  
  
  
  
  
  
  
**3.1.5 How to change the properties**  
  
BE CAREFULL! DO NOT CHANGE ANYTHING, UNLESS YOU KNOW WHAT YOU ARE DOING!  
To change the properties of DNA-Crypt use **Menu->File->Properties**.  
You can change the binary encryption by the first four rows and   
the maximum file size and the maximum genome size by changing the last two rows.   
The MAX\_FILE\_SIZE and MAX\_GENOME\_SIZE must be between 0 and 2147483647.  
The default values are   
00 -> u  
01 -> g  
10 -> c  
11 -> a  
MAX\_FILE\_SIZE : 102400  
MAX\_GENOME\_SIZE : 102400  
  
To reset the properties press the 'Default'-button.  
  
  
  
  
  
  
**3.1.6 How to exit DNA-Crypt**  
  
To exit DNA-Crypt use **Menu->File->Exit**   
or the mnemonic **CTRL+X**.

  
  
Previous - Next
