# Supplementary material for: DNA-based watermarks using the DNA-Crypt algorithm
Source: BMC Bioinformatics. 2007 May 29;8:176. doi: 10.1186/1471-2105-8-176 (PMC1904243; doi:10.1186/1471-2105-8-176)
Supplement: Additional file 1 — The DNA-Crypt v.2. [file 1471-2105-8-176-S1.zip › help/help32.html]

DNA-Crypt  
  
3. The menus

**3.2 The User-Menu**  
  
**3.2.1 How to register a new user**  
  
To register a new user use **Menu->User->Register**.  
Now you have to enter in the information about your person.   
Your username and your password are later used for login to DNA-Crypt.
  
  
  
  
  
  
  
**3.2.2 How to login**  
  
To login to DNA-Crypt, use **Menu->User->Login** and enter your username and password.  
  
  
  
  
  
  
**3.2.3 How to logout**  
  
To logout use **Menu->User->Logout**.  
  
  
  
  
**3.2.4 How to show/change your details**  
  
To show/change your details use **Menu->User->Details**.  
If you want to save the changes to your details press the 'Save'-button.   
Otherwise the 'Cancel'-button.

  
  
Previous - Next
