# Supplementary material for: DNA-based watermarks using the DNA-Crypt algorithm
Source: BMC Bioinformatics. 2007 May 29;8:176. doi: 10.1186/1471-2105-8-176 (PMC1904243; doi:10.1186/1471-2105-8-176)
Supplement: Additional file 1 — The DNA-Crypt v.2. [file 1471-2105-8-176-S1.zip › help/help33.html]

DNA-Crypt  
  
3. The menus

**3.3 The Genome-Menu**  
  
**3.3.1 How to empty the genome**  
  
To empty the genome panel use **Menu->Genome->New**.  
  
  
  
  
  
  
**3.3.2 How to open a genome**  
  
To open a genome use **Menu->Genome->Open...**.  
  
  
  
  
**3.3.3 How to analyse a genome**  
  
To analyse a genome use **Menu->Genome->Analyse**.  
After selecting analyse, you will see the count of each amino acid,  
the count of variable amino acids and the possible letters (Clelland) or bytes,   
which can be encrypted in the genome.  
  
  
  
  
  
  
**3.3.4 How to transcribe a genome**  
  
To transcribe a DNA sequence to RNA use **Menu->Genome->Transcribe**.  
  
  
  
  
**3.3.5 How to translate a genome**  
  
To translate a RNA sequence to a proteine sequence   
use **Menu->Genome->Translate**.  
  
  
  
  
**3.3.6 How to reverse transcribe a genome**  
  
To reverse transcribe a RNA sequence to DNA   
use **Menu->Genome->Reverse**.

  
  
Previous - Next
