# Supplementary material for: DNA-based watermarks using the DNA-Crypt algorithm
Source: BMC Bioinformatics. 2007 May 29;8:176. doi: 10.1186/1471-2105-8-176 (PMC1904243; doi:10.1186/1471-2105-8-176)
Supplement: Additional file 1 — The DNA-Crypt v.2. [file 1471-2105-8-176-S1.zip › help/help34.html]

DNA-Crypt  
  
3. The menus

**3.4 The Key-Menu**  
  
**3.4.1 How to generate new keys**  
  
To generate new keys use **Menu->Key->KeyGenerator**.  
You will have to choose what kind of key you want to create.  
  
  
  
  
  
  
**3.4.2 How to import/export or delete keys**  
  
To import/export or delete keys use **Menu->Key->KeyManager**.  
You can choose a key and export it to file system or import a new key   
to your keylist. You can also delete a key by pressing
the 'Delete'-button.

  
  
Previous - Next
