# Supplementary material for: DNA-based watermarks using the DNA-Crypt algorithm
Source: BMC Bioinformatics. 2007 May 29;8:176. doi: 10.1186/1471-2105-8-176 (PMC1904243; doi:10.1186/1471-2105-8-176)
Supplement: Additional file 1 — The DNA-Crypt v.2. [file 1471-2105-8-176-S1.zip › help/index.html]

DNA-Crypt  
  
  

|  |  |
| --- | --- |
| Index  1. Introduction  2. First steps  3. The menus       3.1 The File-Menu       3.2 The User-Menu       3.3 The Genome-Menu       3.4 The Key-Menu  4. Java Documentation What's new in DNA-Crypt v.2    Download DNA-Crypt v.1 (zip)  Download DNA-Crypt v.2 (zip)  Download fuzzy controller (jar) |  |

  
