# Supplementary material for: DNA-based watermarks using the DNA-Crypt algorithm
Source: BMC Bioinformatics. 2007 May 29;8:176. doi: 10.1186/1471-2105-8-176 (PMC1904243; doi:10.1186/1471-2105-8-176)
Supplement: Additional file 1 — The DNA-Crypt v.2. [file 1471-2105-8-176-S1.zip › help/new.html]

DNA-Crypt v.2  
  
What's new?

There are some extensions in DNA-Crypt v.2:  
  
1. The WDH-Code   
The WDH-Code is another correction code for binary encryption with DNA-Crypt. You can choose whether to use the Hamming Code, the WDH-Code or no mutation correction.   
The WDH-Constant is the count of repeats of the sequence. It should be an odd number.  
  
  
  
  
  
2. Fuzzy controller  
The fuzzy controller decides whether to use the 8/4 Hamming-code, the WDH-Code or no
mutation correction for optimal performance for a given DNA sequence. The life time means the count of generations the watermark should remain without any errors.

  
  
BACK
